# Causal effect of polyunsaturated fatty acids on bone mineral density and fracture

**DOI:** 10.3389/fnut.2022.1014847

**Published:** 2022-12-08

**Authors:** Sha-Sha Tao, Peng Wang, Xin-Yi Wang, Kang-Jia Yin, Xiao-Ke Yang, Zhi-Xin Wang, De-Guang Wang, Hai-Feng Pan

**Affiliations:** ^1^Department of Epidemiology and Biostatistics, School of Public Health, Anhui Medical University, Hefei, Anhui, China; ^2^Inflammation and Immune Mediated Diseases Laboratory of Anhui Province, Hefei, Anhui, China; ^3^Experimental Teaching Center for Preventive Medicine, School of Public Health, Anhui Medical University, Hefei, Anhui, China; ^4^Teaching Center for Preventive Medicine, School of Public Health, Anhui Medical University, Hefei, Anhui, China; ^5^Department of Radiation Oncology, The First Affiliated Hospital of Anhui Medical University, Hefei, Anhui, China; ^6^The First Clinical Medical College, Anhui Medical University, Hefei, Anhui, China; ^7^Department of Rheumatology and Immunology, The First Affiliated Hospital of Anhui Medical University, Hefei, China; ^8^Department of Nephrology, The Second Affiliated Hospital of Anhui Medical University, Hefei, Anhui, China

**Keywords:** polyunsaturated fatty acids, osteoporosis, bone mineral density, genetic association, causal relationship, Mendelian randomization

## Abstract

**Background:**

Polyunsaturated fatty acids (PUFAs) are closely related to osteoporosis. To test their causal relationship, we conducted a Mendelian randomization (MR) analysis.

**Methods:**

We analyzed the causal relationship between four PUFAs measures, n-3 PUFAs (n-3), n-6 PUFAs (n-6), the ratio of n-3 PUFAs to total fatty acids (n-3 pct), and the ratio of n-6 PUFAs to n-3 PUFAs (n-6 to n-3), and five measures of osteoporosis, including estimated bone mineral density (eBMD), forearm (FA) BMD, femoral neck (FN) BMD, lumbar spine (LS) BMD, and fracture, using two-sample MR analysis. In order to verify the direct effect between PUFAs and BMD, we chose interleukin-6 (IL-6), tumor necrosis factor-β (TNF-β), and bone morphogenetic proteins 7 (BMP-7), three markers or cytokines strongly related to BMD, as possible confounding factors, and analyzed the possible causal relationships between them and PUFAs or BMD by MR. Inverse variance weighting (IVW), MR-Egger, weighted and weighted median were conducted. MR Pleiotropy RESidual Sum and Outlier (MR-PRESSO) and MR-Egger regression methods were used to evaluate the potential pleiotropy of instrumental variables (IVs) and outliers were identified by MR-PRESSO. Cochran’s Q statistic was used to detect the heterogeneity among IVs. Leave-one-out sensitivity analysis was used to find SNPs that have a significant impact on the results. All results were corrected by the Bonferroni correction.

**Results:**

The IVW results showed that n-3 PUFAs (OR = 1.030, 95% CI: 1.013, 1.047, *P* = 0.001) and n-6 PUFAs (OR = 1.053, 95% CI: 1.034, 1.072, *P* < 0.001) were positively correlated with eBMD, while n-6 to n-3 (OR = 0.947, 95% CI: 0.924, 0.970, *P* < 0.001) were negatively correlated with eBMD. These casual relationships still existed after Bonferroni correction. There were positive effects of n-3 PUFAs on FA BMD (OR = 1.090, 95% CI: 1.011, 1.176, *P* = 0.025) and LS BMD (OR = 1.056, 95% CI: 1.011, 1.104, *P* = 0.014), n-3 pct on eBMD (OR = 1.028, 95% CI: 1.002, 1.055, *P* = 0.035) and FA BMD (OR = 1.090, 95% CI: 1.011, 1.174, *P* = 0.025), n-6 to n-3 on LS BMD (OR = 1.071, 95% CI: 1.021, 1.124, *P* = 0.005); negative effects of n-3 pct on fracture (OR = 0.953, 95% CI: 0.918, 0.988, *P* = 0.009) and n-6 to n-3 on FA BMD (OR = 0.910, 95% CI: 0.837, 0.988, *P* = 0.025). However, these causal effects all disappeared after Bonferroni correction (all *P* > 0.0025). None of IL-6, TNF-β, and BMP-7 had a causal effect on PUFA and BMD simultaneously (all *P* > 0.05).

**Conclusion:**

Evidence from this MR study supports the genetically predicted causal effects of n-3, n-6, n-3 pct, and n-6 to n-3 on eBMD. In addition, n-3 not only associate with FA BMD and LS BMD through its own level and n-6 to n-3, but also link to fracture through n-3 pct.

## Introduction

Osteoporosis is one of the most common systemic bone diseases in clinic, which is mainly manifested with the degradation of bone tissue microenvironment, the reduction of bone mineral density (BMD), the increase of bone fragility, and the high risk of fracture (1, 2). Osteoporosis mostly occurs in the postmenopausal women. Worldwide, fractures caused by osteoporosis can be as high as 9 million times a year (3). With the increased life expectancy of the global population and the improvement of medical and health conditions, the prevention and treatment of osteoporosis and fractures is still a common public health challenge and health care problem in the world today (4–7). It has been suggested that 20–40% of the risk of osteoporosis is caused by environmental factors such as nutrition (8).

Polyunsaturated fatty acids (PUFAs) are important immune nutrients, which play a role in nutritional treatment of a variety of diseases, including cancers, inflammatory diseases and osteoporosis (9–20). PUFAs are straight chain fatty acids with two or more double bonds, and the length of carbon chain is 18–22 carbon atoms. In PUFAs, the first unsaturated double bond is located between the 3rd and 4th carbon atoms starting from the methyl end, which is called omega-3 (n-3) PUFAs, and between the 6th and 7th carbon atoms, which is called omega-6 (n-6) PUFAs (15). Mounting evidence has suggested that PUFAs are involved in osteolysis, bone formation, bone development, bone metabolism, and metabolic bone diseases including osteoporosis and may be beneficial for skeletal health (21–29).

Recently, the relationship between PUFAs and osteoporosis has attracted a lot of attention. PUFAs intake and the ratio of n-6 to n-3 PUFAs are reported to be associated with BMD in humans (27, 30–32). Some studies found that total PUFAs, n-3 PUFAs, n-6 PUFAs intake can increase BMD, decrease the risk of fractures and beneficial for osteoporosis (30–34). A low n-6 to n-3 PUFAs ratio was proposed to be beneficial for the bone quality of rats (35). However, conclusions about the relationship between n-3 PUFAs, n-6 PUFAs, and osteoporosis are inconsistent. It was also observed that the intake of n-3 PUFAs or the ratio of n-6 to n-3 PUFAs are not associated with osteoporotic fractures, while the intake of n-6 PUFAs is positively associated with an elevated risk of fracture (32). Other study also indicated that increased intake of PUFAs is associated with greater perimenopausal femoral neck (FN) BMD loss (36). In postmenopausal women, the lower intake of marine n-3 PUFAs and the higher intake of n-6 PUFAs were observed that can decrease the risk of total fracture (33).

Clarifying the relationship between various PUFAs levels and BMD can not only further study the prevention, health care and treatment strategies of osteoporosis patients, but also can estimate the fracture risk of osteoporosis patients. It is imperative to evaluate the causal relationship between PUFAs level and BMD. However, from the above evidence, conclusions from observational studies and randomized controlled trials (RCT) about the relationship between PUFAs and osteoporosis are inconsistent, as well as the causal relationship between PUFAs and osteoporosis remains obscure.

Genome wide association study (GWAS) shows that BMD is a trait controlled by multiple genes, and is easy to be affected by environmental factors and has the tendency of family aggregation. Some genetic determinants of fractures are regulated by lower BMD (37–40). Mendelian randomization (MR) is an advanced research using the law of free combination, which uses genetic variation as instrumental variable (IVs) (41). The free combination of alleles can reduce the confounding effect of environmental factors; the one-way relationship that genes affect traits but diseases do not change genotypes can effectively avoid reverse causal bias (42). Compared with RCT, MR can extract useful information from the existing GWAS database on a large scale, with stronger statistical ability and wider coverage (43).

## Materials and methods

### Study design

In this study, we used two-sample MR studies to assess the causal relationships between PUFAs and osteoporosis. We selected four measures of PUFAs, including the circulating level of n-3 PUFAs (n-3), the circulating level of n-6 PUFAs (n-6), the ratio of n-3 fatty acids to total fatty acids (n-3 pct), and the ratio of n-6 PUFAs to n-3 PUFAs (n-6 to n-3). For osteoporosis, estimated BMD (eBMD), forearm (FA) BMD, femoral neck (FN) BMD, lumbar (LS) BMD, and fracture were selected as measures.

In order to verify the direct effect between PUFAs and BMD, we chose interleukin-6 (IL-6) (44), tumor necrosis factor-β (TNF-β) (45) and bone morphogenetic proteins 7 (BMP-7) (46), three markers or cytokines strongly related to BMD, as possible confounding factors, and analyzed the possible causal relationships between them and PUFAs or BMD by MR.

### Data sources and single-nucleotide polymorphism selection

#### Genome wide association study of polyunsaturated fatty acids

The GWAS data on PUFAs came from the latest and largest public GWAS analysis by Borges MC et al. (47). In the study, the GWAS data of n-3, n-6, n-3 pct, and n-6 to n-3 were generated from 114,999 UK Biobank participants of European ancestry using BOLT-LMM (v2.3) (Supplementary Table 1) (48).

#### Genome wide association studyof osteoporosis

Genetic associations of eBMD and fracture were obtained from the largest public GWAS of Morris et al. (1), in which eBMD GWAS data were retrieved from 426,824 individuals of European ancestry in the UK Biobank and fracture GWAS data were retrieved from 416,795 UK Biobank European ancestry participants (ncases = 53,184 and ncontrols = 373,611) (Supplementary Table 1).

The research by Zheng et al. (49) provided GWAS data about FA BMD, FN BMD, and LS BMD. In the research, the GWAS data for the three BMDs were obtained from 10,805, 49,988, and 44,731 individuals of European ancestry, respectively (Supplementary Table 1).

#### Genome wide association study of possible confounding factors

Genetic associations of IL-6 and TNF-β were obtained from the public GWAS of Ahola-Olli et al. (50), and the GWAS data for both were retrieved from 8,293 individuals of European ancestry. The GWAS data of BMP-7 was obtained from 3,301 individuals of European ancestry (51) (Supplementary Table 1).

#### Single-nucleotide polymorphism selection

We screened single-nucleotide polymorphisms (SNPs) strongly related to exposure factors (*P* < 5 × 10^–8^) from the exposure GWAS, used clustering process (*R*^2^ < 0.001 and clumping distance = 10,000 kb) to eliminate linkage disequilibrium (LD) between SNPs, and excluded SNPs with minor allele frequency (MAF < 0.01), which ensured that the end result was undisturbed and feasible. The selected SNPs were matched with the outcome GWAS, and if SNP can not be found in outcome GWAS, its proxy SNP with high LD (*r*^2^ > 0.8) was used instead. Finally, other SNPs were selected as IVs after the palindrome SNPs were removed.

### Statistical analyses

We used four complementary and mutually corroborative methods to analyze the causal relationship between PUFAs and osteoporosis, including inverse variance weighting (IVW), MR-Egger, weighted and weighted median, among which IVW is the main analysis method. The weighted median estimator serves as an unbiased causal effect estimate when up to 50% of the instruments are invalid, by estimating the causal effect as the median of the weighted ratio estimates (52). At the same time, we used MR Pleiotropy RESidual Sum and Outlier (MR-PRESSO) and MR-Egger regression methods to evaluate the potential level pleiotropy of IVs (53, 54). Meanwhile, MR-PRESSO can also find abnormal values in IVs. After removing the abnormal values, MR-PRESSO and MR-Egger tests were performed again until there was no horizontal pleiotropic SNP in all IVs. Then, we applied Cochran’s Q statistic to detect and quantify the heterogeneity among IVs (55). Leave-one-out sensitivity analysis was used to find and eliminate SNPs that have a significant impact on the results, so as to ensure the robustness of causal relationship estimation. There were four exposures (n-3, n-6, n-3pct, and n-6 to n-3) and five outcomes (eBMD, FA, FN, LS, and fracture) in this study, therefore the Bonferroni method was conducted to correct for multiple comparisons and the *P*-value was less than 0.0025 (0.05 was divided by 4 × 5) (56, 57). All statistical analyses were performed using the packages “TwoSampleMR” and “MRPRESSO” in R version 4.1.1.

## Results

### Instrumental variables selection

#### Instrumental variables of polyunsaturated fatty acids selection

After clumping process, there were 52 SNPs, 63 SNPs, 41 SNPs, and 41 SNPs strongly associated (*P* < 5 × 10^–8^) with the circulating level of n-3 PUFAs, the circulating level of n-6 PUFAs, n-3 pct, n-6 to n-3, respectively and no LD was screened out. MAF of all above SNPs were not less than 0.01. The main information of SNPs is listed in Supplementary Tables 2–5.

#### Instrumental variables of possible confounding factors selection

After clumping process, there were 2 SNPs and 2 SNPs strongly associated (*P* < 5 × 10^–8^) with TNF-β and BMP-7, respectively. There was no SNPs available at the genome wide significance threshold (*P* < 5 × 10^–8^) of IL-6, so we relaxed the significance threshold to *P* < 5 × 10^–7^, and 2 SNPs were strongly associated (*P* < 5 × 10^–7^) with IL-6. Among the above selected SNPs, no LD was found, and MAF of these SNPs were not less than 0.01. The main information of SNPs is provided in Supplementary Tables 6, 7.

### Causal relationship between polyunsaturated fatty acids and osteoporosis

#### n-3 polyunsaturated fatty acids on osteoporosis

**FIGURE 1:**
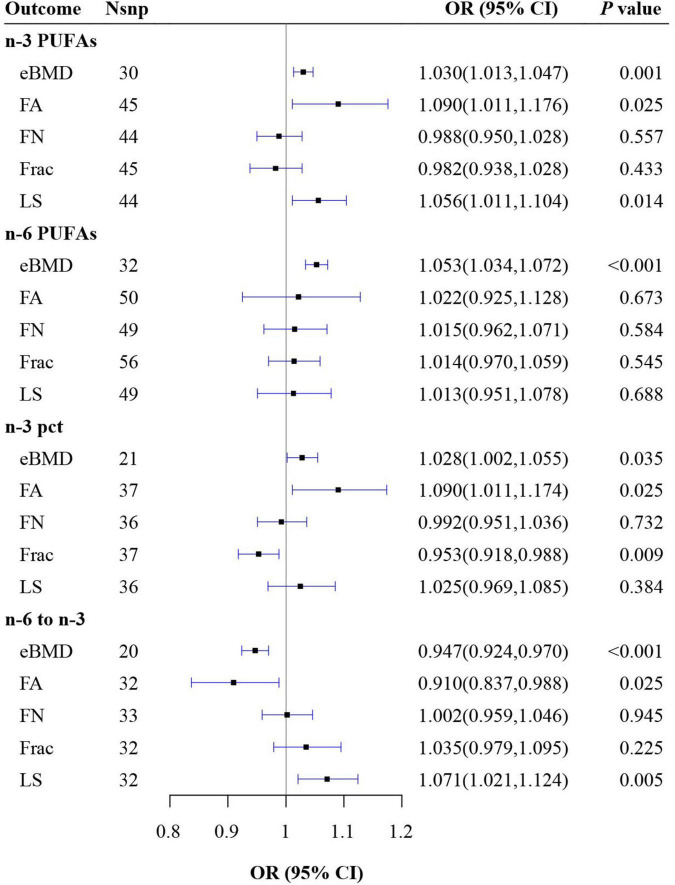
Mendelian randomization (MR) estimate results of polyunsaturated fatty acids (PUFAs) on outcomes. n-3 pct, the ratio of n-3 fatty acids to total fatty acids; n-6 to n-3, the ratio of n-6 PUFAs to n-3 PUFAs; BMD, bone mineral density; eBMD, estimated BMD; FA, forearm; FN, femoral neck; LS, lumbar.

From the IVW results, n-3 PUFAs had positive effects on eBMD (OR = 1.030, 95% CI: 1.013, 1.047, *P* = 0.001), FA BMD (OR = 1.090, 95% CI: 1.011, 1.176, *P* = 0.025), and LS BMD (OR = 1.056, 95% CI: 1.011, 1.104, *P* = 0.014), however, after further Bonferroni correction, only the effect on eBMD remained. No causal effects of n-3 PUFAs on FN BMD (OR = 0.988, 95% CI: 0.950, 1.028, *P* = 0.557) and fracture (OR = 0.982, 95% CI: 0.938, 1.028, *P* = 0.433) were observed (Figure 1 and Supplementary Table 8).

#### n-6 polyunsaturated fatty acids on osteoporosis

The results of IVW showed a positive effect of n-6 PUFAs on eBMD (OR = 1.053, 95% CI: 1.034, 1.072, *P* < 0.001), which still persisted after Bonferroni correction. However, no causal associations were found between n-6 PUFAs and FA BMD (OR = 1.022, 95% CI: 0.925, 1.128, *P* = 0.673), FN BMD (OR = 1.015, 95% CI: 0.962, 1.071, *P* = 0.584), LS BMD (OR = 1.013, 95% CI: 0.951, 1.078, *P* = 0.688), and fracture (OR = 1.014, 95% CI: 0.970, 1.059, *P* = 0.545) (Figure 1 and Supplementary Table 9).

#### n-3 pct on osteoporosis

The results of IVW revealed that n-3 pct had positive causal relationships with eBMD (OR = 1.028, 95% CI: 1.002, 1.055, *P* = 0.035) and FA BMD (OR = 1.090, 95% CI: 1.011, 1.174, *P* = 0.025) as well as a negative causal relationship with fracture (OR = 0.953, 95% CI: 0.918, 0.988, *P* = 0.009). However, these causal effects all disappeared after Bonferroni correction. There were no causal effects of n-3 pct on FN BMD (OR = 0.992, 95% CI: 0.951, 1.036, *P* = 0.732) and LS BMD (OR = 1.025, 95% CI: 0.969, 1.085, *P* = 0.384) (Figure 1 and Supplementary Table 10).

#### n-6 to n-3 on osteoporosis

The results of IVW indicated that n-6 to n-3 had a negative effect on eBMD (OR = 0.947, 95% CI: 0.924, 0.970, *P* < 0.001), which remain persisted after Bonferroni correction and had a negative relationship with FA BMD (OR = 0.910, 95% CI: 0.837, 0.988, *P* = 0.025) while a positive relationship with LS BMD (OR = 1.071, 95% CI: 1.021, 1.124, *P* = 0.005), which both disappeared after Bonferroni correction. No causal effects of n-6 to n-3 on FN BMD (OR = 1.002, 95% CI: 0.959, 1.046, *P* = 0.945) and fracture (OR = 1.035, 95% CI: 0.979, 1.095, *P* = 0.225) were observed (Figure 1 and Supplementary Table 11).

### Causal relationship between possible confounding factors and polyunsaturated fatty acids

**FIGURE 2:**
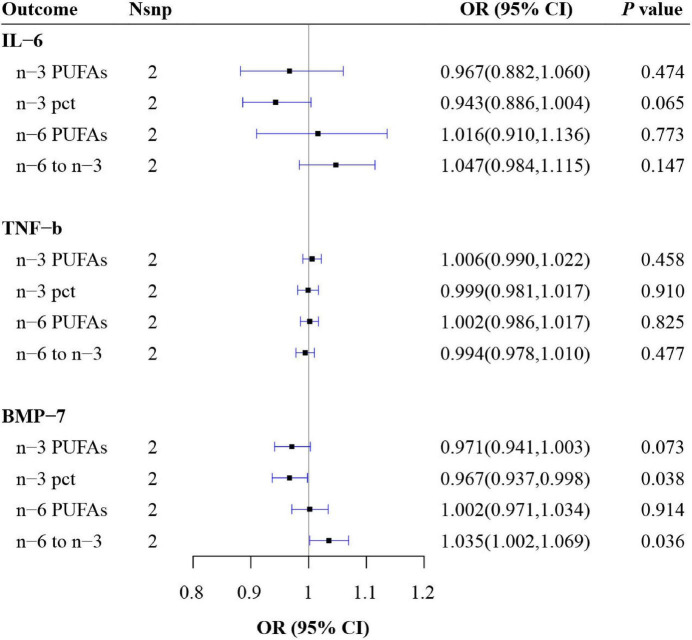
Mendelian randomization (MR) estimate results of possible confounding factors on polyunsaturated fatty acids (PUFAs). n-3 pct, the ratio of n-3 fatty acids to total fatty acids; n-6 to n-3, the ratio of n-6 PUFAs to n-3 PUFAs; IL, interleukin; TNF, tumor necrosis factor; BMP, bone morphogenetic protein.

From the IVW results, BMP-7 had a negative effect on n-3 pct (OR = 0.967, 95% CI: 0.937, 0.998, *P* = 0.038) and a positive effect on n-6 to n-3 (OR = 1.035, 95% CI: 1.002, 1.069, *P* = 0.036). However, no causal effect was observed of BMP-7 on n-3 PUFAs and n-6 PUFAs (all *P* > 0.05). Moreover, no causal relationship was found between IL-6, TNF-β and all four outcomes (all *P* > 0.05) (Figure 2 and Supplementary Table 12).

### Causal relationship between possible confounding factors and osteoporosis

**FIGURE 3:**
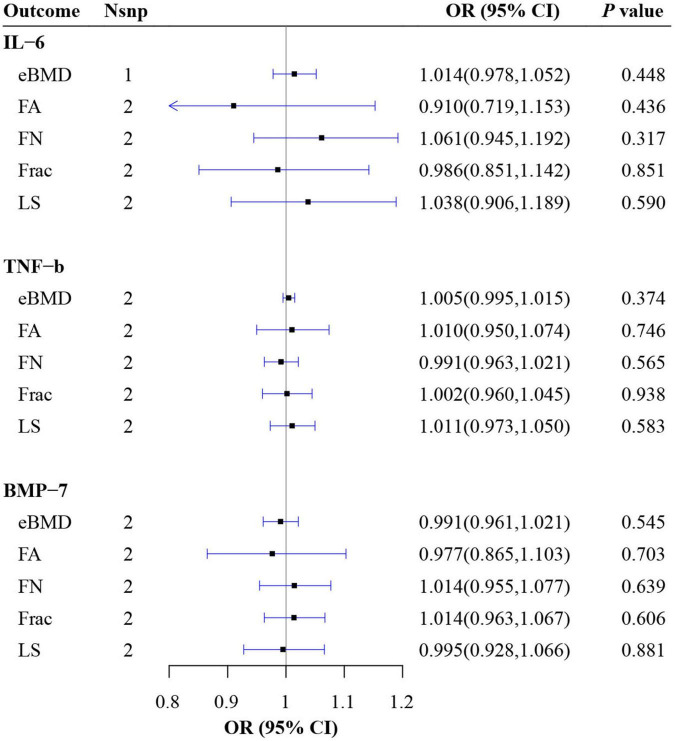
Mendelian randomization (MR) estimate results of possible confounding factors on BMD. BMD, bone mineral density; eBMD, estimated BMD; FA, forearm; FN, femoral neck; LS, lumbar; IL, interleukin; TNF, tumor necrosis factor; BMP, bone morphogenetic protein.

From the IVW results, no causal relationship was found between IL-6, TNF-β, BMP-7 and eBMD, FA, FN, LS as well as fracture (all *P* > 0.05) (Figure 3 and Supplementary Table 13).

### Pleiotropy and sensitivity analysis

The heterogeneity test did not find any heterogeneity between selected IVs of n-3 PUFAs and n-6 PUFAs. Heterogeneity was not observed between the IVs of n-3 pct on eBMD and on FA BMD, while was found among IVs of n-3 pct on FN BMD, on fractrue and on LS BMD. No heterogeneities were found between IVs of n-6 to n-3, except for IVs of n-6 to n-3 on FN BMD (Q = 50.008, *P* = 0.022). No heterogeneity was found between the IVs of IL-6, TNF-β and BMP-7.

MR Pleiotropy RESidual Sum and Outlier global test defined SNPs with horizontal pleiotropy as outliers which were listed in Supplementary Tables 2–7. After removing the outliers, MR-Egger regression and MR-PRESSO global test were used to verify that there was no horizontal pleiotropy between IVs and results. Leave-one-out analysis suggested that the outcomes were not caused by any SNPs. Supplementary Tables 8–13 and Supplementary Figures 1–16 show the results of pleiotropy and sensitivity analysis.

## Discussion

As the IVW results shown, after Bonferroni correction n-3 PUFAs and n-6 PUFAs were still positively correlated with eBMD, while n-6 to n-3 were negatively correlated with eBMD, which provides new evidence to support the relationship between PUFAs and osteoporosis.

In this study, no causal effect was observed of IL-6 and TNF-β on four measures of PUFAs and five measures of osteoporosis, suggesting that the causal effect of four measures of PUFAs on BMD was not affected by IL-6 and TNF-β, moreover IL-6 and TNF-β could not affect BMD by affecting PUFAs. As we all know, only in situations where the total effect, direct effect and indirect effect all act in the same direction, the exposure having indirect effect can be identified as a mediator (58). BMP-7 has a causal effect on-3 pct and n-6 to n-3, but has no causal effect on BMD, suggesting that BMP-7 can not affect BMD through PUFAs and is also not a mediator in the causal effect of PUFAs on BMD.

Recently, the relationship between PUFAs and osteoporosis has attracted a lot of attention. Some studies have found that intake of total PUFA, n-3 PUFAs, n-6 PUFAs can increase BMD and be beneficial to osteoporosis (30–34), which is mutually confirmed with our results.

These may attribute to the profitable role of PUFAs in bone formation, absorption, development and metabolism, and n-3 PUFAs can also regulate bone health by increasing osteoblast activity and decreasing osteoclast activity, promoting intestinal calcium absorption and mineral deposition during bone development (25). The intake of n-3 PUFAs has also been observed to be associated with increased bone regeneration, improved bone microstructure and strength (59–61). Osteoporosis increases the apoptosis, adipogenic differentiation, and levels of RANKL and sclerostin of bone marrow mesenchymal stem cells and osteoblasts (62). Bone mineral loss is the result of an imbalance between osteoblastic bone formation and osteoclast bone resorption. As an important n-3 PUFAs, DHA is a lipid component specific to the osteoblast membrane, which induces extensive lipid remodeling in mesenchymal stem cells, resulting in more stable membrane microdomains and thus enhanced osteogenic differentiation (63). It has been reported that dietary n-3 can reduce osteoclast formation and bone loss in ovariectomized mice (64). In rats, taking fish oil can also inhibit alveolar bone absorption and osteoclast activity (65).

In this study, n-6 to n-3 was observed to be negatively related to eBMD, which was consistent with previous studies. In previous study, a low n-6 to n-3 PUFAs ratio has also been proposed to be beneficial for the bone quality of rats (35). It has been widely documented that reducing the n-6 to n-3 PUFAs ratio can prevent bone mineral loss and prostaglandin(PG) E2 production in animal and *in vitro* cell culture experiments (66). Kelly et al. (67) also proposed that the high proportion of n-6 to n-3 PUFAs may be one of the important reasons for the increased risk of obesity and osteoporosis. According to the IVW results, the relationship between n-3 PUFAs, n-6 PUFAs, or the ratio of n-6 to n-3 PUFAs and fractures was not observed, while n-3 pct was negatively related to fractures, which suggests that it may not be the level of PUFAs who affected the fracture, but n-3 pct. These results, together with the n-6 to n-3 results, suggest that n-3 may influence BMD not only by itself or by its ratio to n-6, but also by its ratio to total fatty acids. The single item has little significance, but its proportion has important diagnostic value, which is not uncommon in clinical practice. For example, neutrophils to lymphocytes ratio (NLR) is an inflammatory index, which plays a role in the prognosis evaluation of sepsis and various diseases. A higher NLR may indicate more serious infection and worse prognosis (68, 69). The ratio of soluble fms-like tyrosine kinase 1 (sFlt-1) to placental growth factor (PlGF) can be used as a monitoring indicator of preeclampsia (PE), which is a kind of hypertensive disorder complicating pregnancy and seriously endangers the health of mothers and infants, with unpredictable outcomes (70).

Moreover, the IVW results showed that FA BMD and LS BMD, which were less affected by weight bearing, were more susceptible to n-3 and n-6 to n-3 than FN BMD which was more affected by weight bearing. Fat is digested, decomposed and metabolized into glycerin and PUFAs (71). Like vitamins and minerals, PUFAs are closely related to bone health through various ways. The distribution and accumulation of adipose tissue is extremely important for bone health. At the same time, adipose tissue secretions such as leptin, adiponectin, estrogen and osteocalcin can also act on bones. Multiple studies have shown that BMI is positively correlated with BMD, with lower BMI being thought to increase the risk of osteoporosis, while higher body weight (even obesity) protects bones (72–75). However, in recent years, this “obesity paradox” has been challenged like never before. Fat-rich bone marrow may be the cause of osteoporosis, especially in postmenopausal women (76). In 2011, the UK Fracture Liaison Service first reported that the incidence of obesity in postmenopausal women with fragility fractures can be as high as 27% (77). Two other studies were also pointed out that visceral fat was significantly associated with bone loss (78, 79). Excessive body fat, especially abdominal fat, produces inflammatory cytokines that stimulate increased bone marrow lipogenesis, increased bone resorption, decreased bone strength, and decreased bone mass (80).

## Advantages and disadvantages

This study has several advantages. First of all, as a MR study, this study investigated the causal associations between different types of PUFAs and BMD in different parts, with detailed classification and comprehensive research. Secondly, we not only studied the level of PUFAs, but also the proportion of PUFAs, and all the results verified each other, and the conclusions were unified. Third, we used a strict Bonferroni correction, thus the conclusions are robust.

At the same time, this study also has some limitations. First, all GWAS data in this study were from European populations, and the representativeness of the results to the entire population remains to be determined. Second, the relationship between different doses of PUFAs and BMD has not been studied, and more detailed quantitative experiments are needed. In addition, the mediating effects of obesity, BMD and other factors still need further research.

## Conclusion

This MR study establishes that n-3, n-6, n-3 pct, and n-6 to n-3 are causally associated with eBMD. In addition, n-3 not only associated with FA BMD and LS BMD through its own level and n-6 to n-3, but also associated with fracture through n-3 pct. Our findings provide new clue to further reveal the pathogenic role and therapeutic potential of PUFAs in osteoporosis.

## Data availability statement

The original contributions presented in this study are included in the article/Supplementary material, further inquiries can be directed to the corresponding author.

## Author contributions

H-FP and D-GW conceived the present idea and were responsible for the design of the study. S-ST performed the statistical analysis and manuscript writing. PW and X-YW participated in acquisition of data and data analysis. K-JY, X-KY, and Z-XW participated in acquisition of data. All authors have read and approved the submitted version for publication.
