# Supplementary material for: Causal effect of polyunsaturated fatty acids on bone mineral density and fracture
Source: Front Nutr. 2022 Dec 8;9:1014847. doi: 10.3389/fnut.2022.1014847 (PMC9772990; doi:10.3389/fnut.2022.1014847)
Supplement: Supplementary file 1 [file Data_Sheet_1.docx]

**
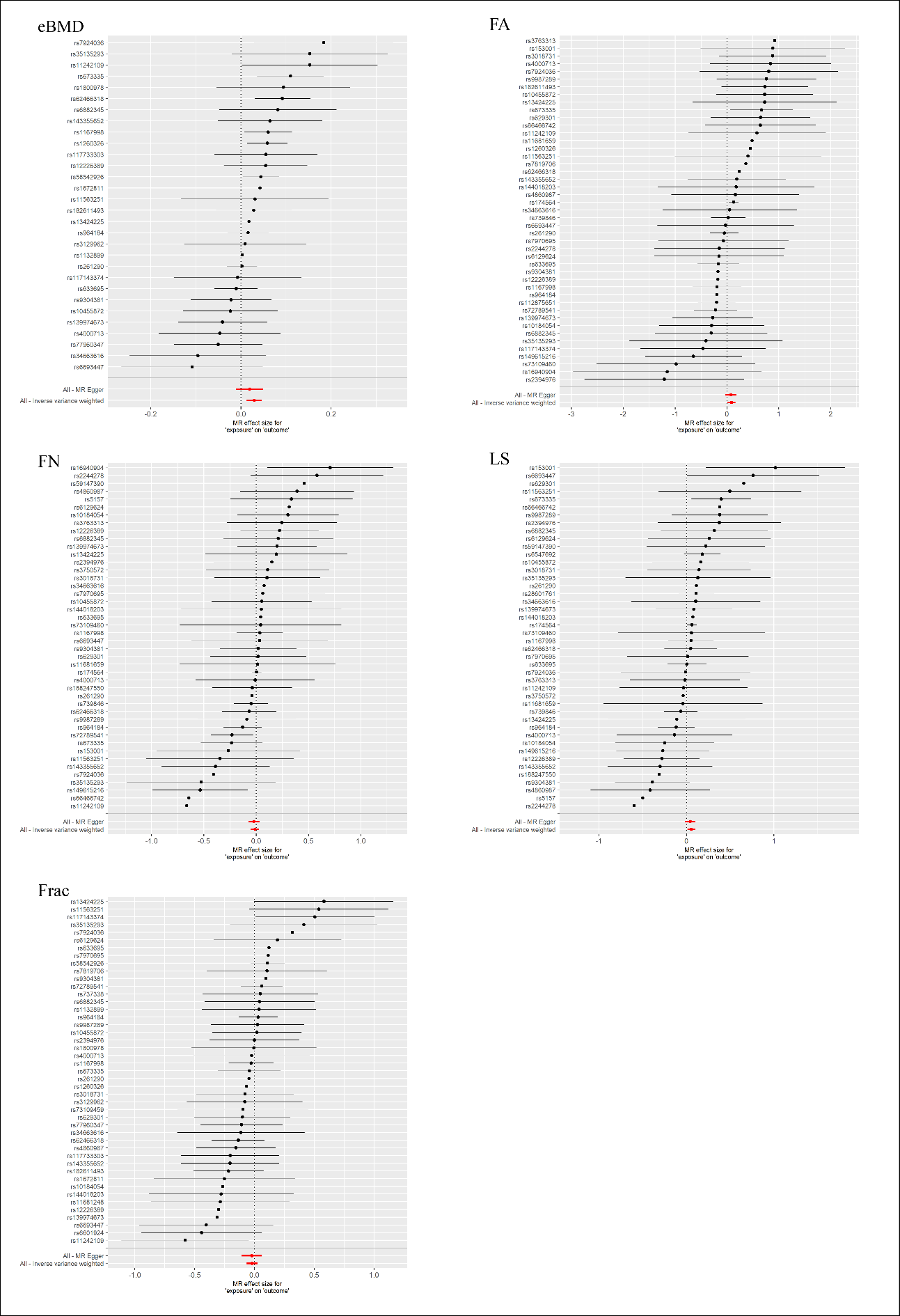
**

**Supplementary Figure 1.** Forest plot of n-3 PUFAs on outcomes.

**Abbreviations:** BMD: bone mineral density; eBMD: estimated BMD; FA: forearm BMD; FN: femoral neck BMD; LS: lumbar BMD; Frac: fracture.


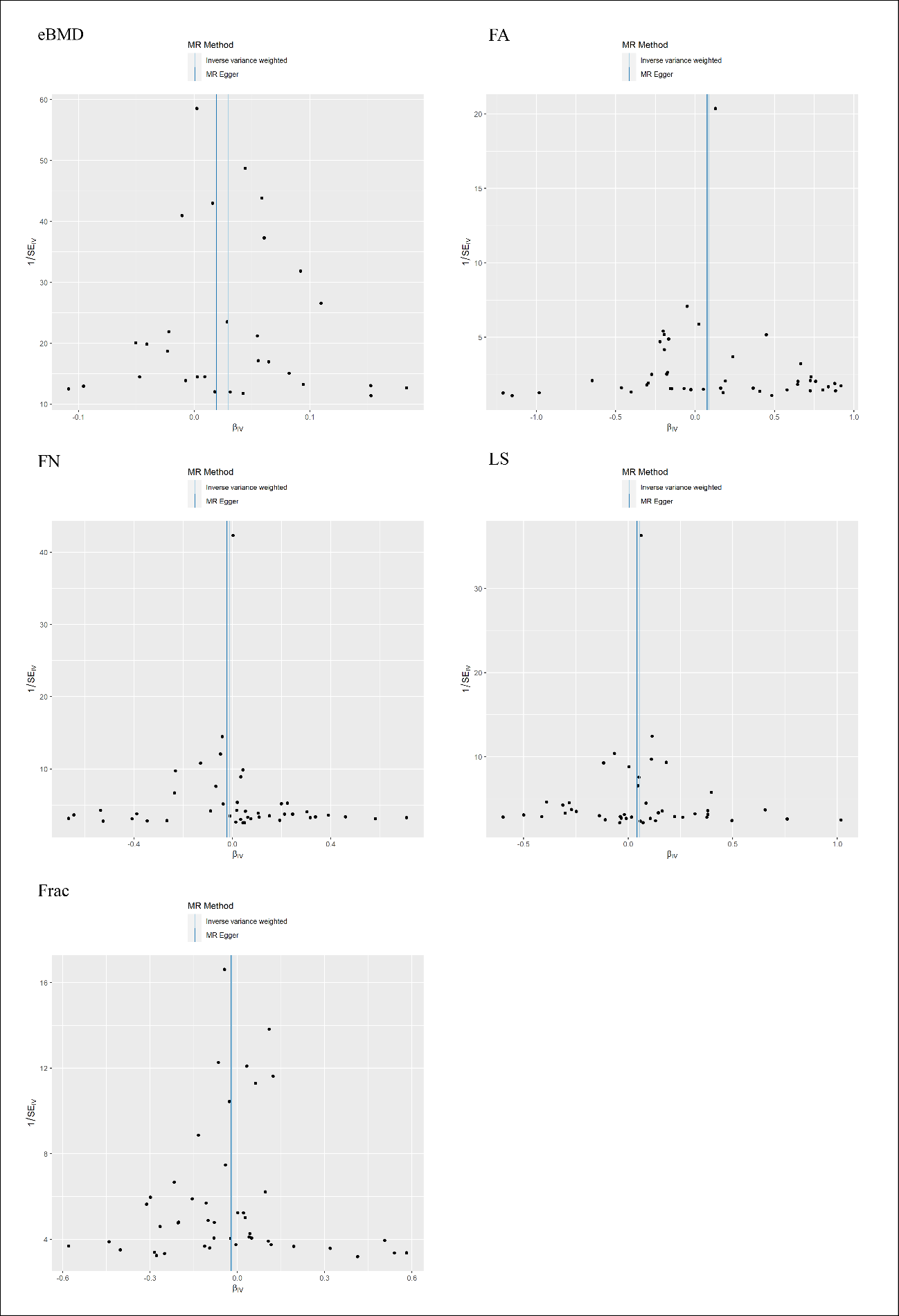


**Supplementary Figure 2.** Funnel plots of n-3 PUFAs on outcomes.

**Abbreviations:** BMD: bone mineral density; eBMD: estimated BMD; FA: forearm BMD; FN: femoral neck BMD; LS: lumbar BMD; Frac: fracture.

**
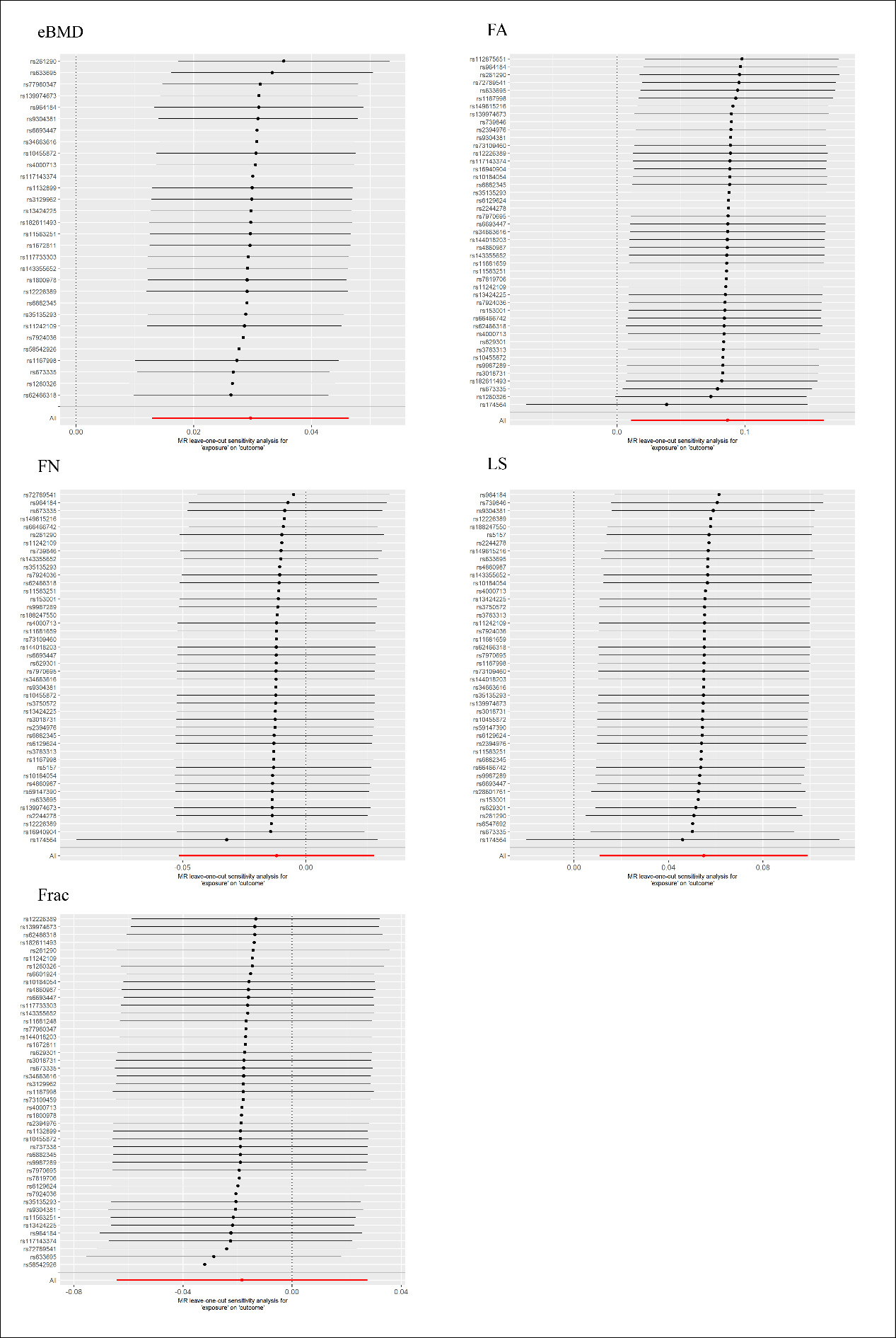
Supplementary Figure 3.** Leave-one-out plots of n-3 PUFAs on outcomes.

**Abbreviations:** BMD: bone mineral density; eBMD: estimated BMD; FA: forearm BMD; FN: femoral neck BMD; LS: lumbar BMD; Frac: fracture.

**
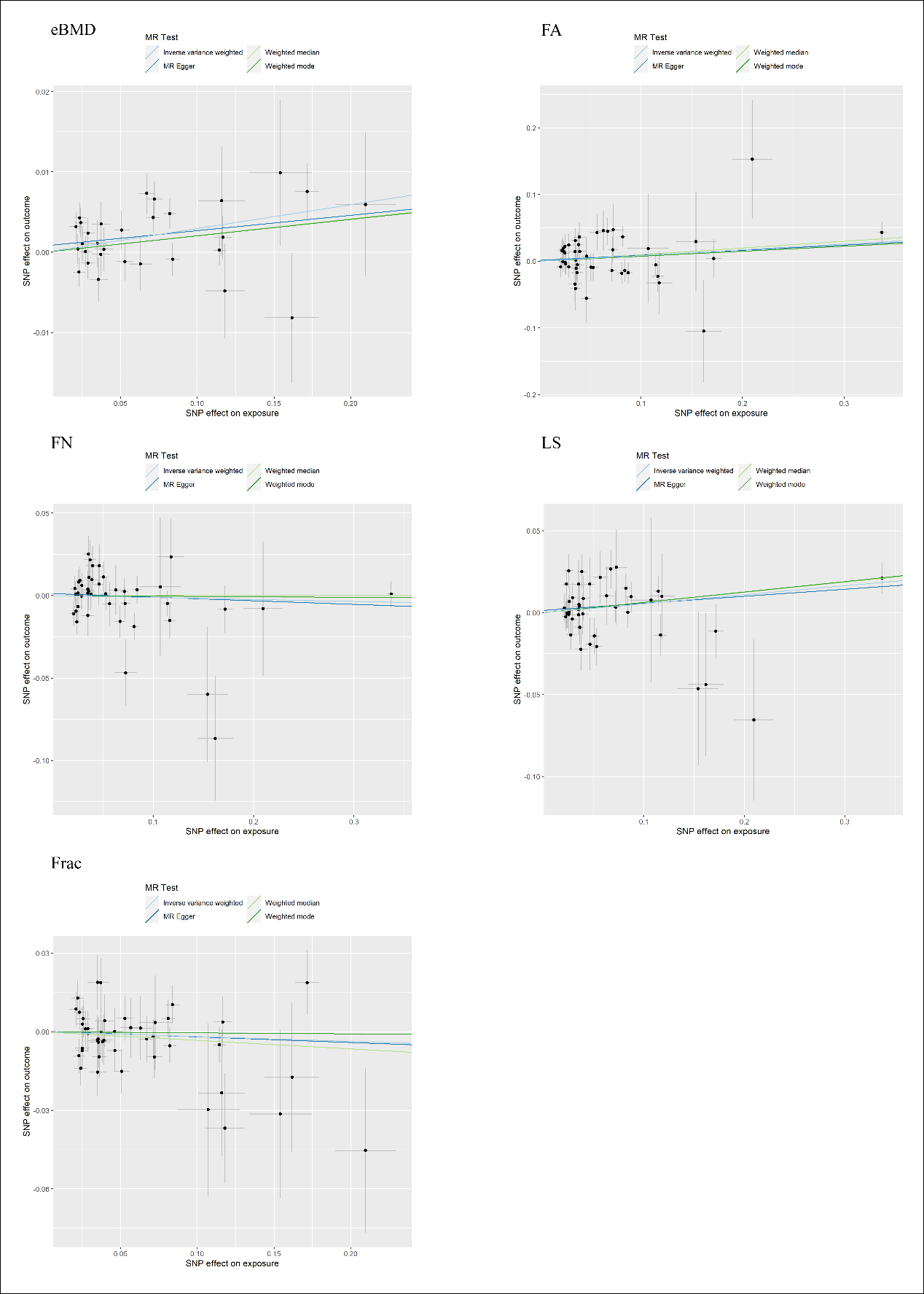
Supplementary Figure 4.** Scatter plots of n-3 PUFAs on outcomes.

**Abbreviations:** BMD: bone mineral density; eBMD: estimated BMD; FA: forearm BMD; FN: femoral neck BMD; LS: lumbar BMD; Frac: fracture.


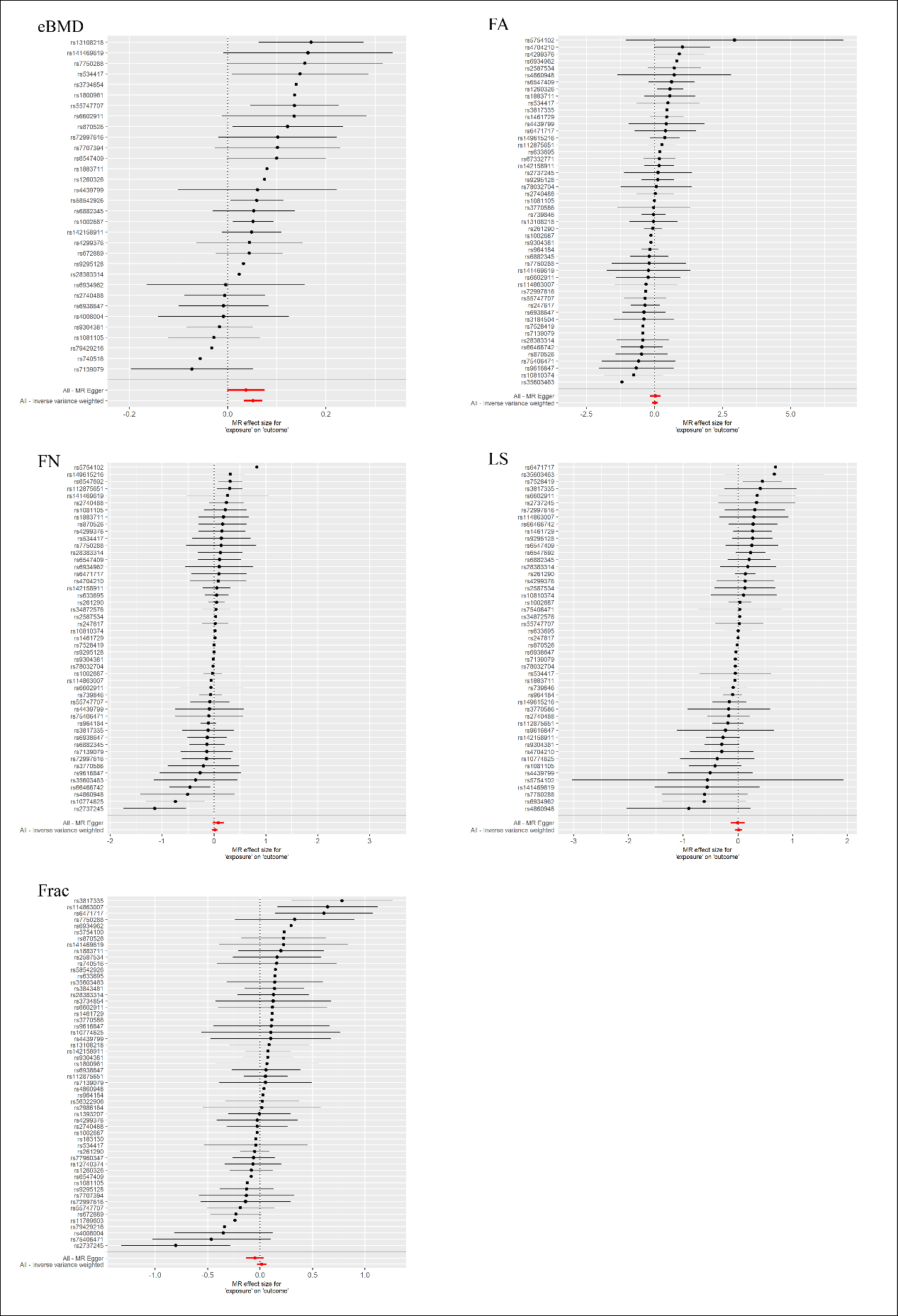


**Supplementary Figure 5.** Forest plots of n-6 PUFAs on outcomes.

**Abbreviations:** BMD: bone mineral density; eBMD: estimated BMD; FA: forearm BMD; FN: femoral neck BMD; LS: lumbar BMD; Frac: fracture.


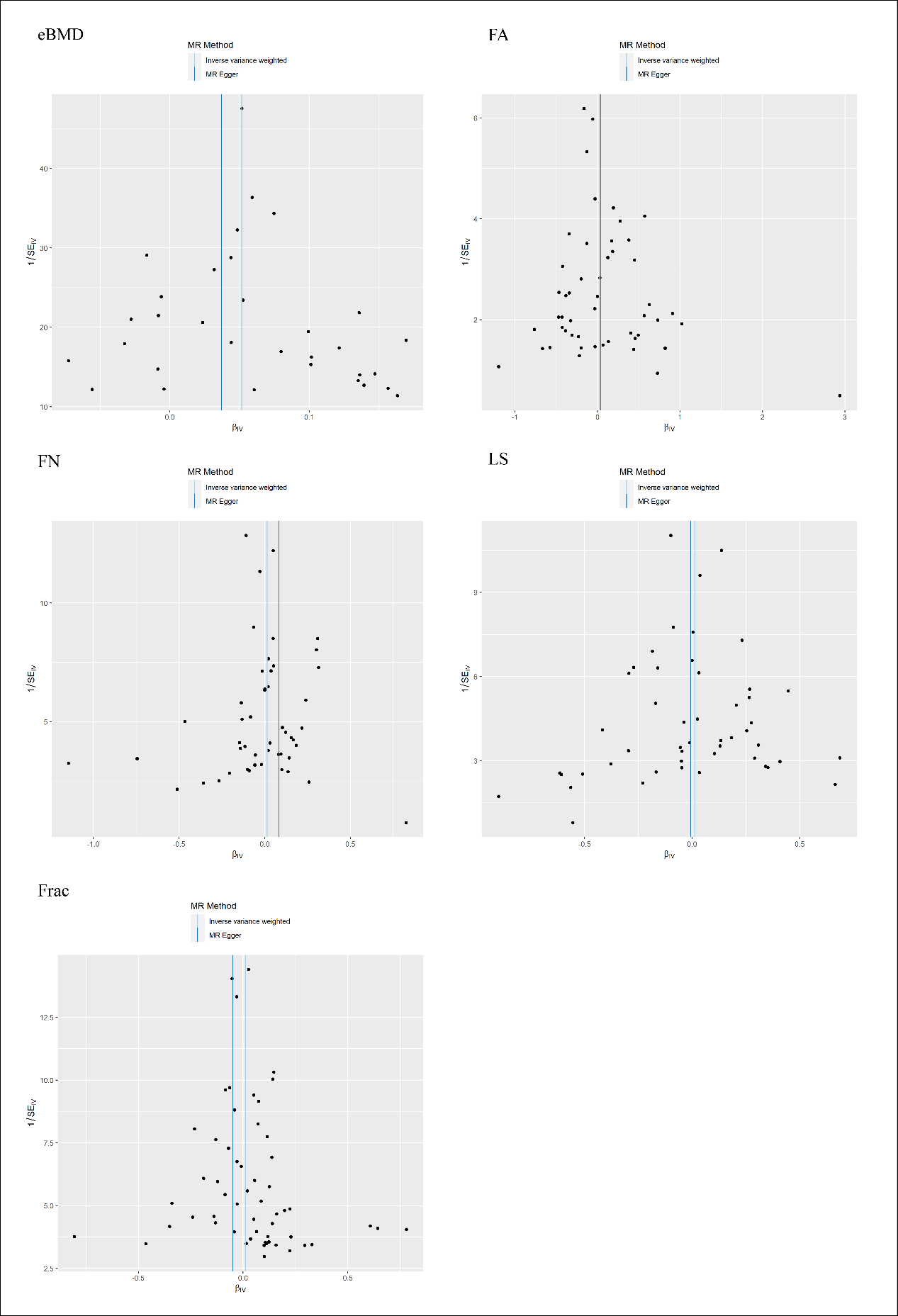


**Supplementary Figure 6.** Funnel plots of n-6 PUFAs on outcomes.

**Abbreviations:** BMD: bone mineral density; eBMD: estimated BMD; FA: forearm BMD; FN: femoral neck BMD; LS: lumbar BMD; Frac: fracture.


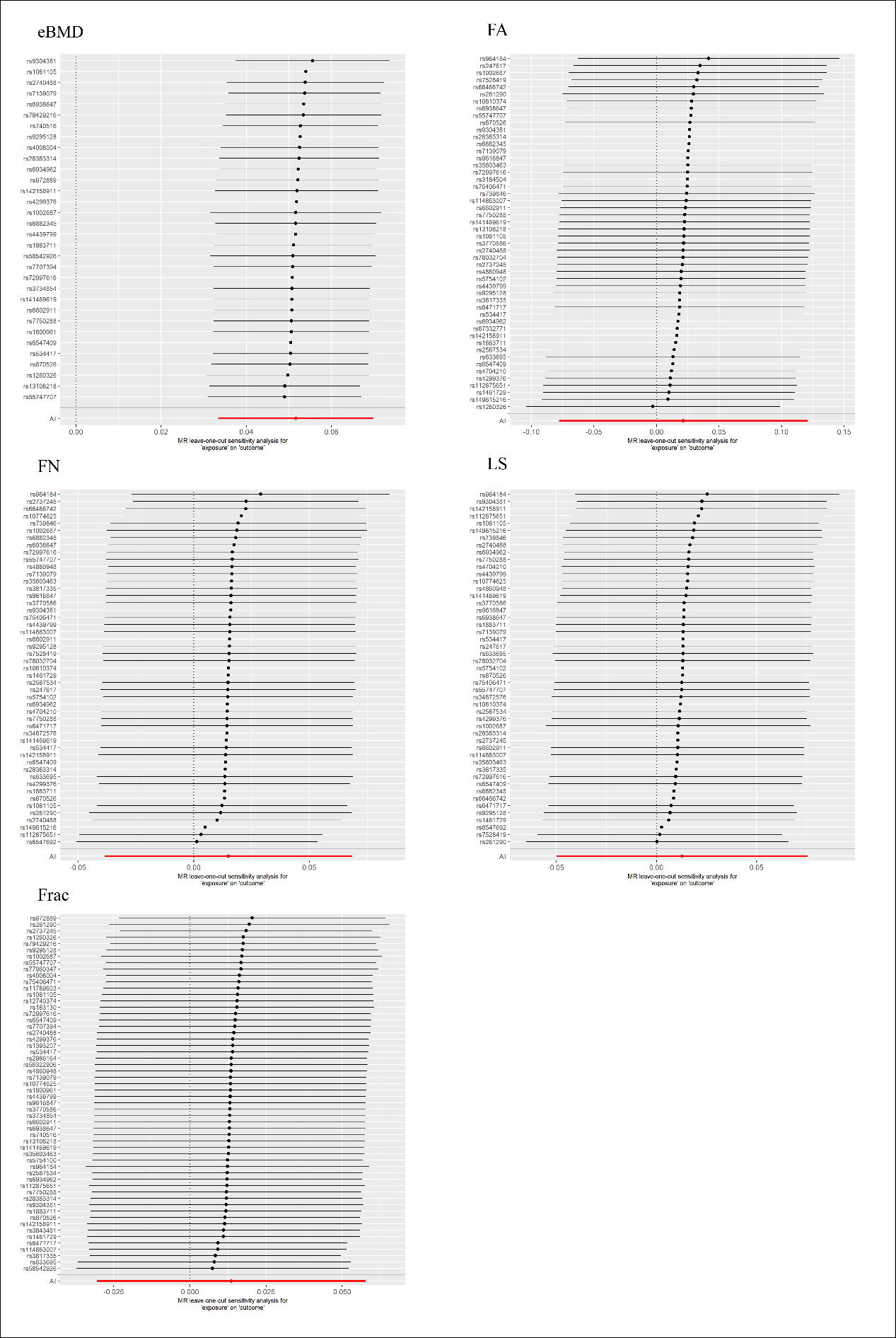


**Supplementary Figure 7.** Leave-one-out plots of n-6 PUFAs on outcomes.

**Abbreviations:** BMD: bone mineral density; eBMD: estimated BMD; FA: forearm BMD; FN: femoral neck BMD; LS: lumbar BMD; Frac: fracture.


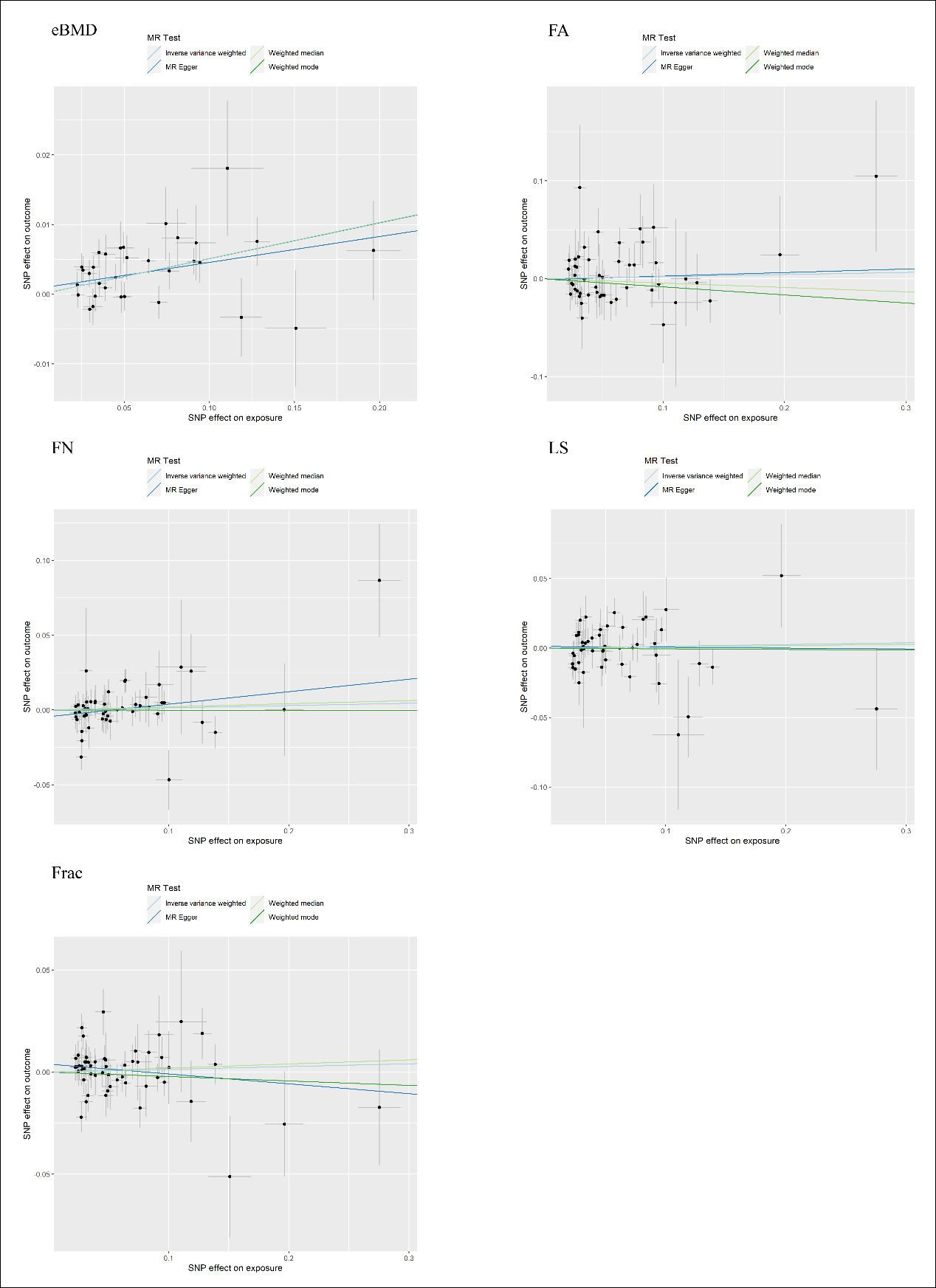


**Supplementary Figure 8.** Scatter plots of n-6 PUFAs on outcomes.

**Abbreviations:** BMD: bone mineral density; eBMD: estimated BMD; FA: forearm BMD; FN: femoral neck BMD; LS: lumbar BMD; Frac: fracture.

**
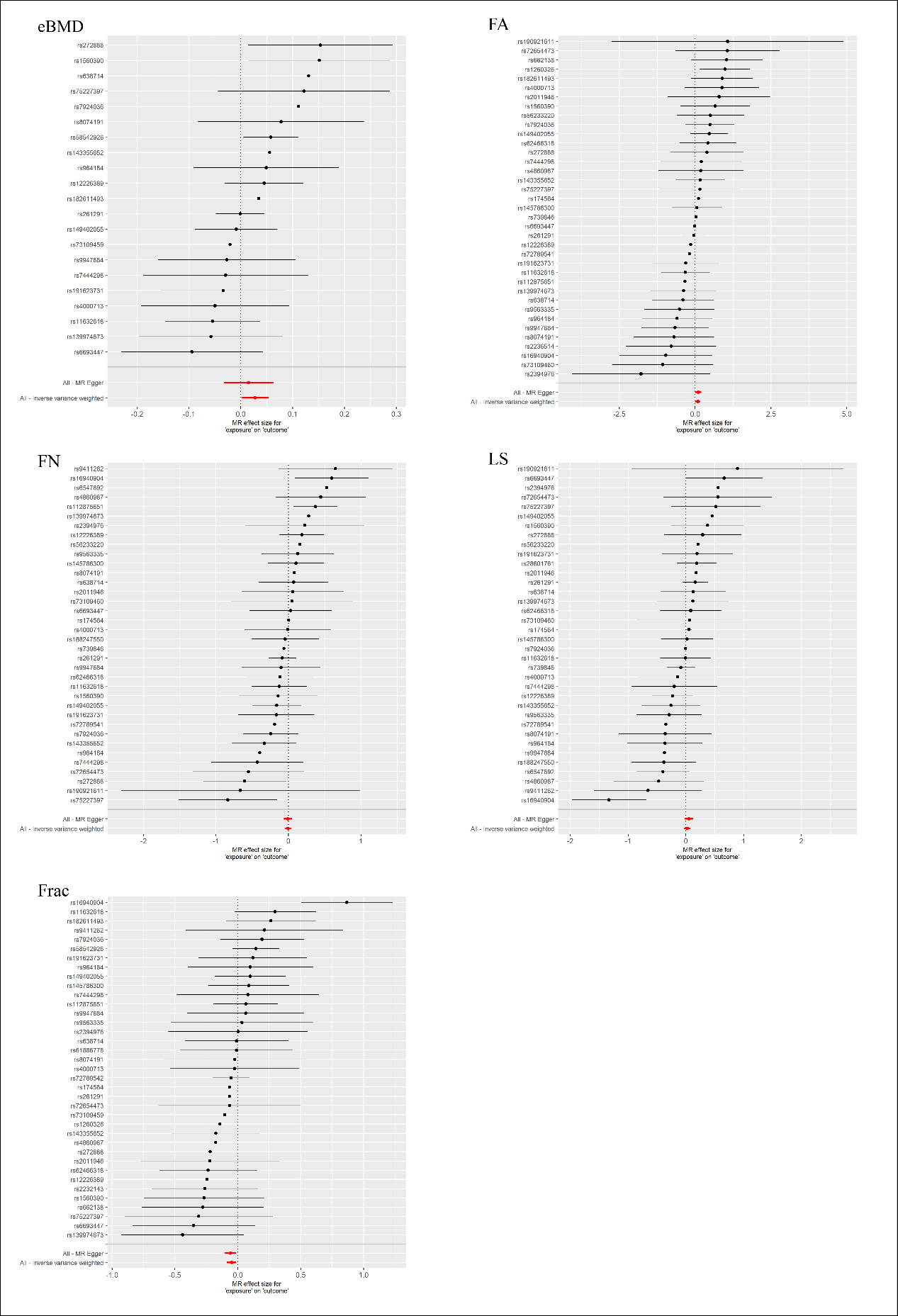
Supplementary Figure 9.** Forest plots of the ratio of n-3 fatty acids to total fatty acids on outcomes.

**Abbreviations:** BMD: bone mineral density; eBMD: estimated BMD; FA: forearm BMD; FN: femoral neck BMD; LS: lumbar BMD; Frac: fracture.


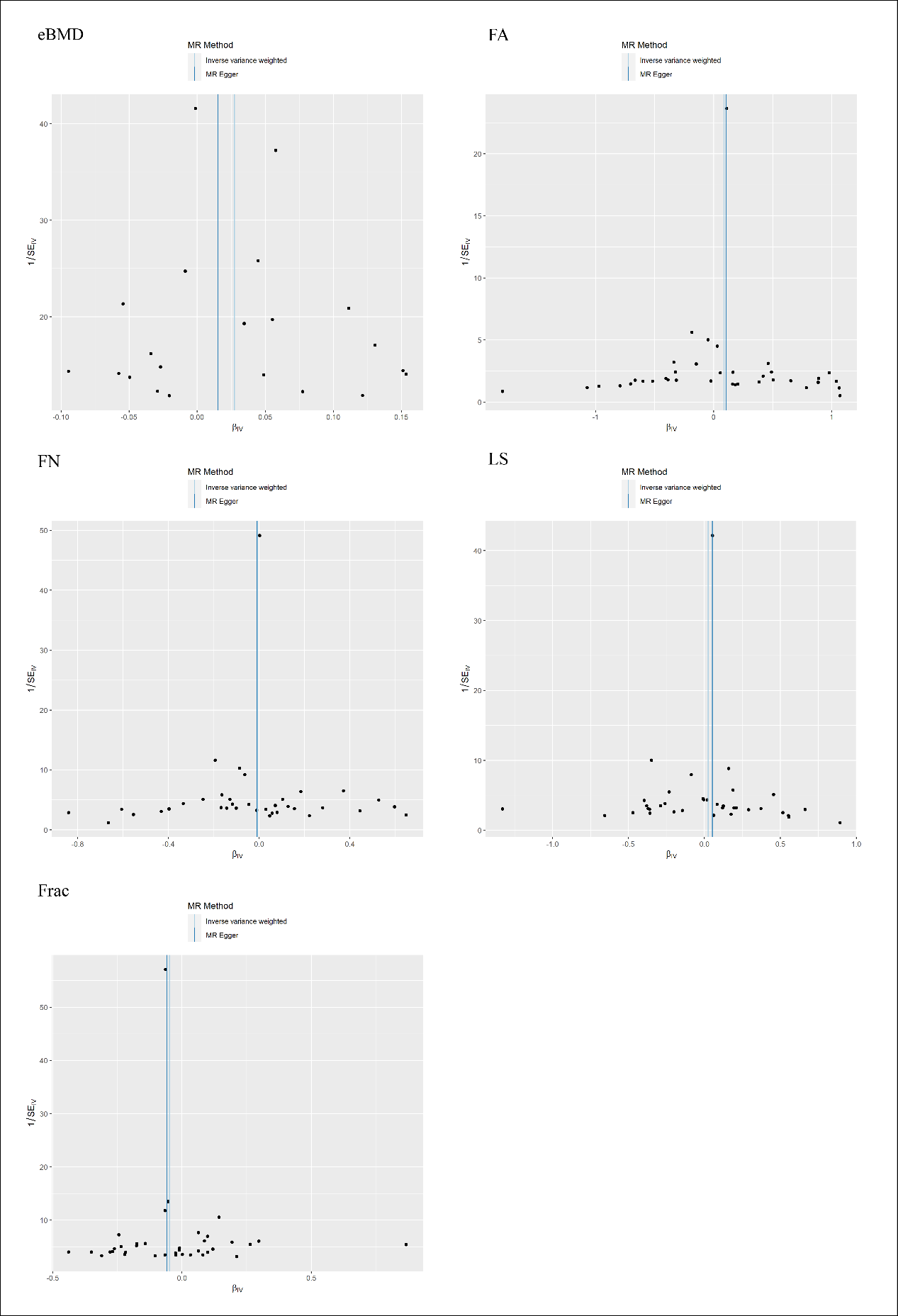


**Supplementary Figure 10.** Funnel plots of the ratio of n-3 fatty acids to total fatty acids on outcomes.

**Abbreviations:** BMD: bone mineral density; eBMD: estimated BMD; FA: forearm BMD; FN: femoral neck BMD; LS: lumbar BMD; Frac: fracture.


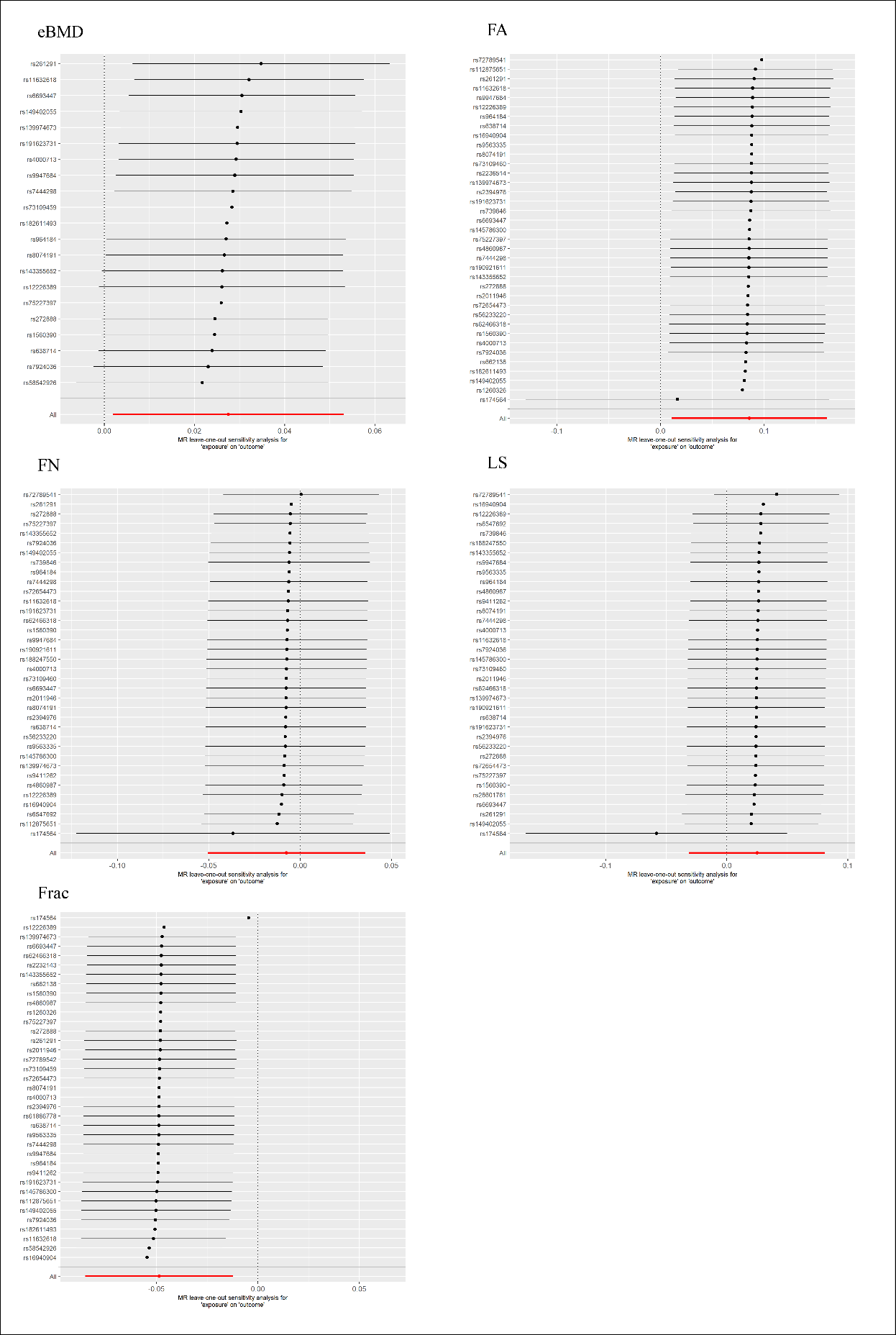


**Supplementary Figure 11.** Leave-one-out plots of the ratio of n-3 fatty acids to total fatty acids on outcomes.

**Abbreviations:** BMD: bone mineral density; eBMD: estimated BMD; FA: forearm BMD; FN: femoral neck BMD; LS: lumbar BMD; Frac: fracture.


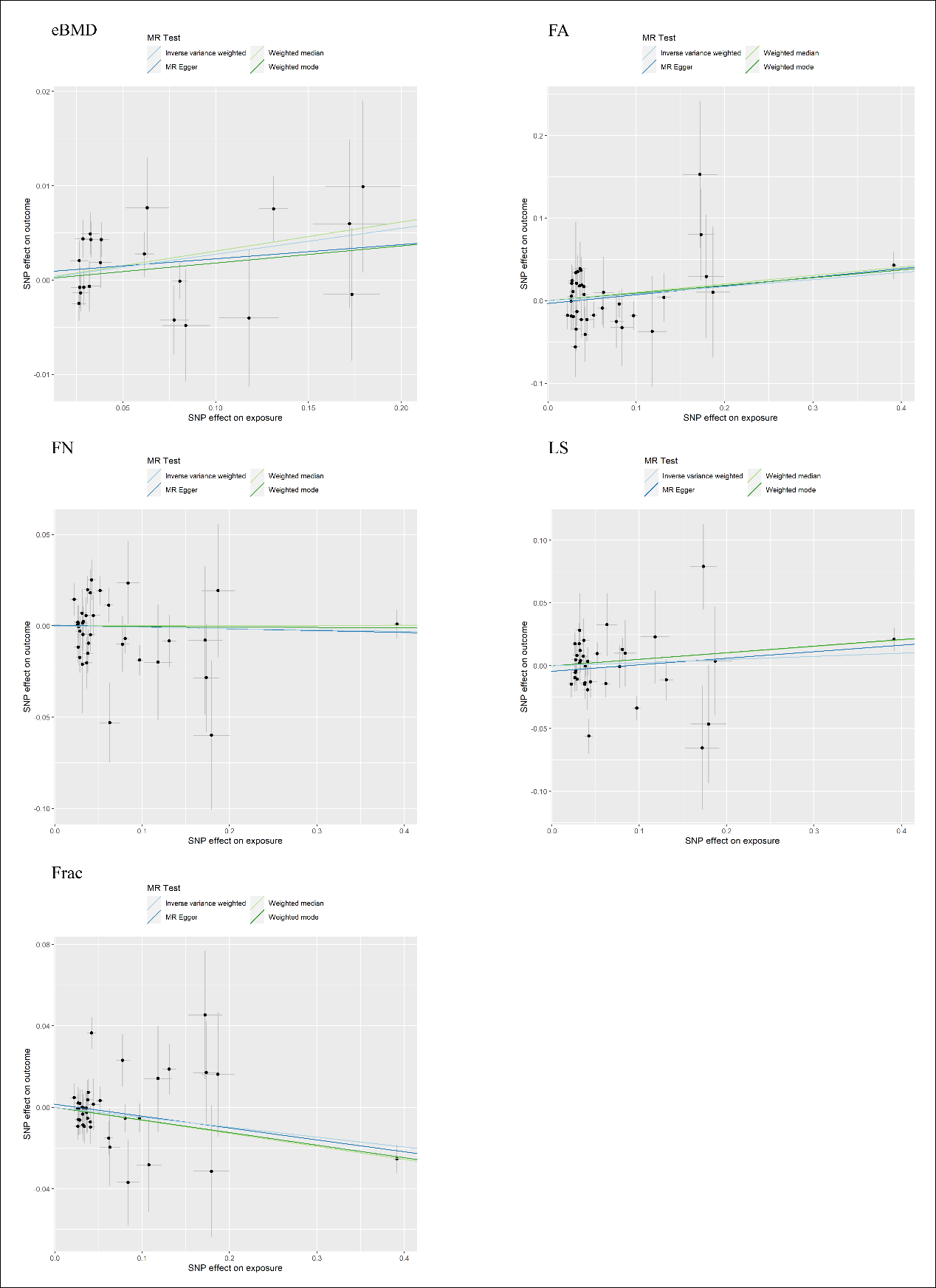


**Supplementary Figure 12.** Scatter plots of the ratio of n-3 fatty acids to total fatty acids on outcomes.

**Abbreviations:** BMD: bone mineral density; eBMD: estimated BMD; FA: forearm BMD; FN: femoral neck BMD; LS: lumbar BMD; Frac: fracture.


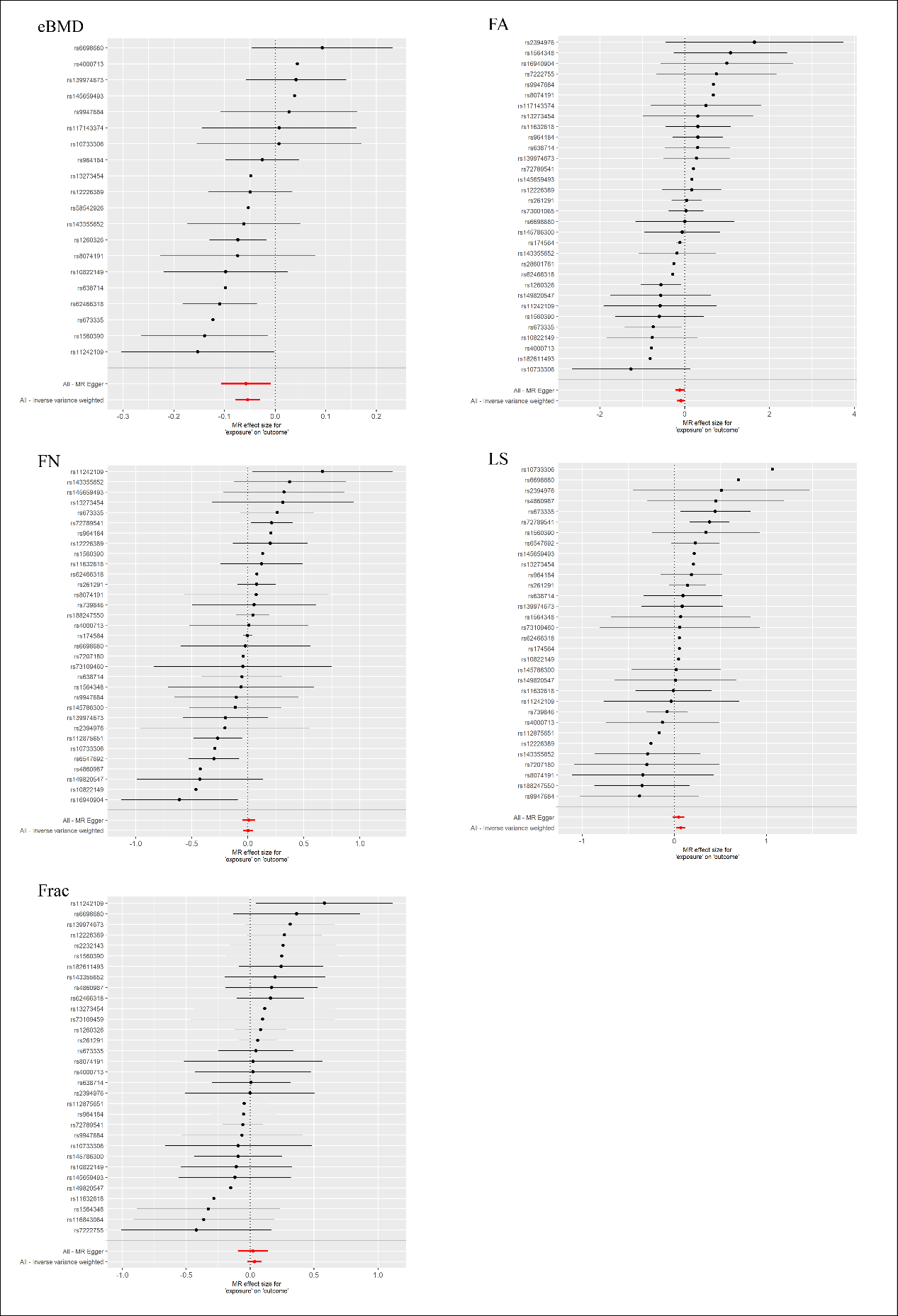


**Supplementary Figure 13.** Forest plots of the ratio of n-6 PUFAs to n-3 PUFAs on outcomes.

**Abbreviations:** BMD: bone mineral density; eBMD: estimated BMD; FA: forearm BMD; FN: femoral neck BMD; LS: lumbar BMD; Frac: fracture.


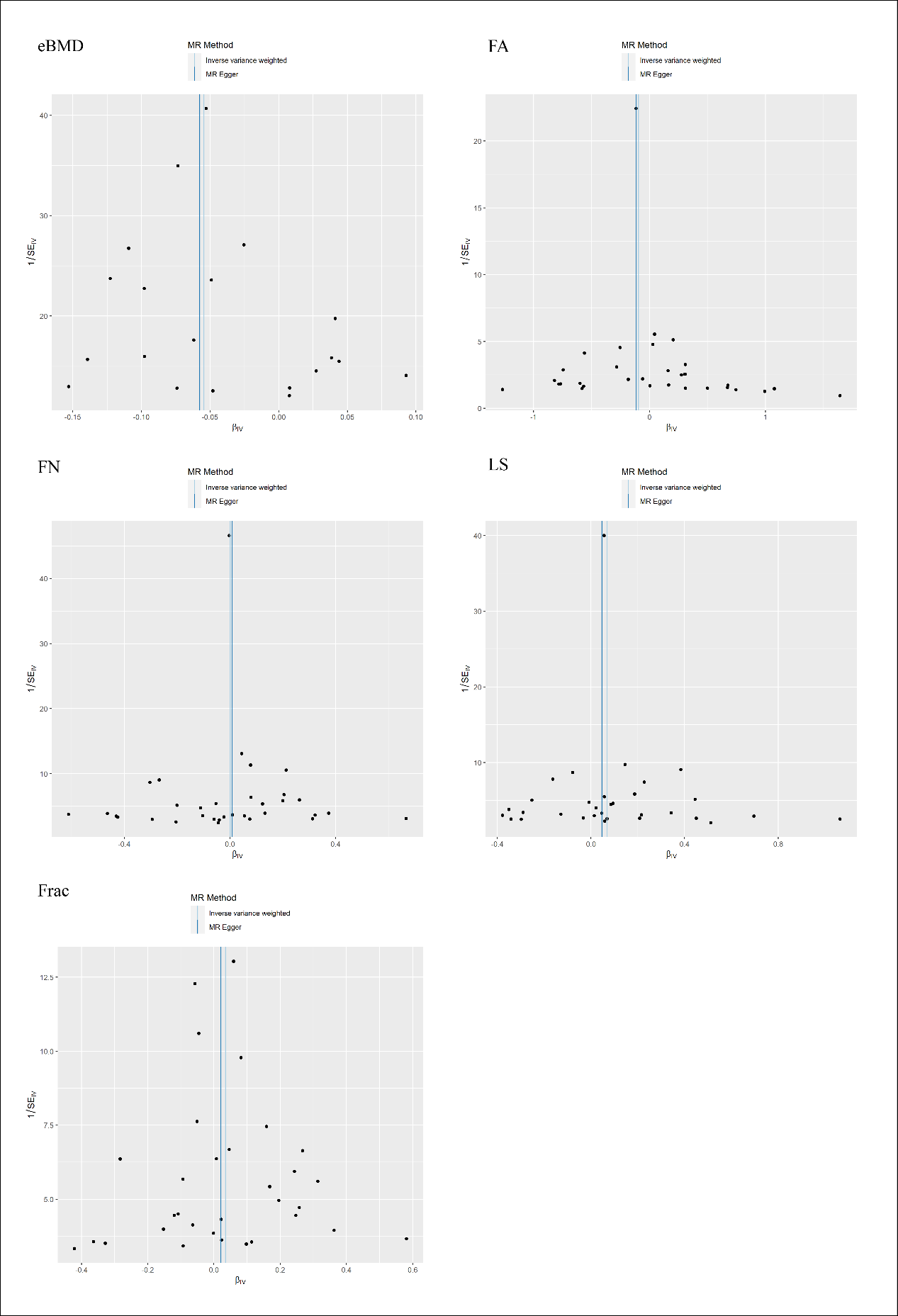


**Supplementary Figure 14.** Funnel plots of the ratio of n-6 PUFAs to n-3 PUFAs on outcomes.

**Abbreviations:** BMD: bone mineral density; eBMD: estimated BMD; FA: forearm BMD; FN: femoral neck BMD; LS: lumbar BMD; Frac: fracture.


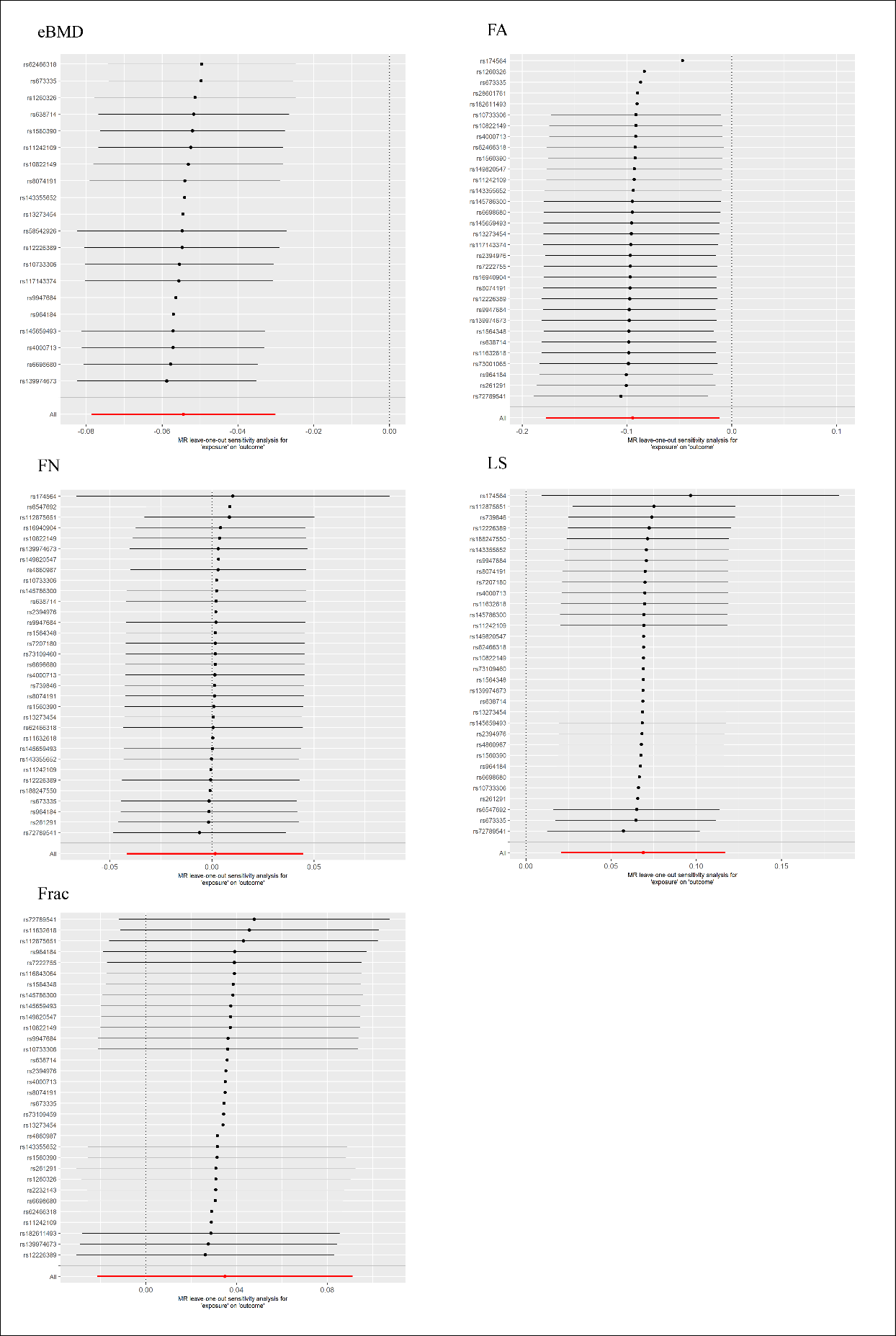


**Supplementary Figure 15.** Leave-one-out plots of the ratio of n-6 PUFAs to n-3 PUFAs on outcomes.

**Abbreviations:** BMD: bone mineral density; eBMD: estimated BMD; FA: forearm BMD; FN: femoral neck BMD; LS: lumbar BMD; Frac: fracture.


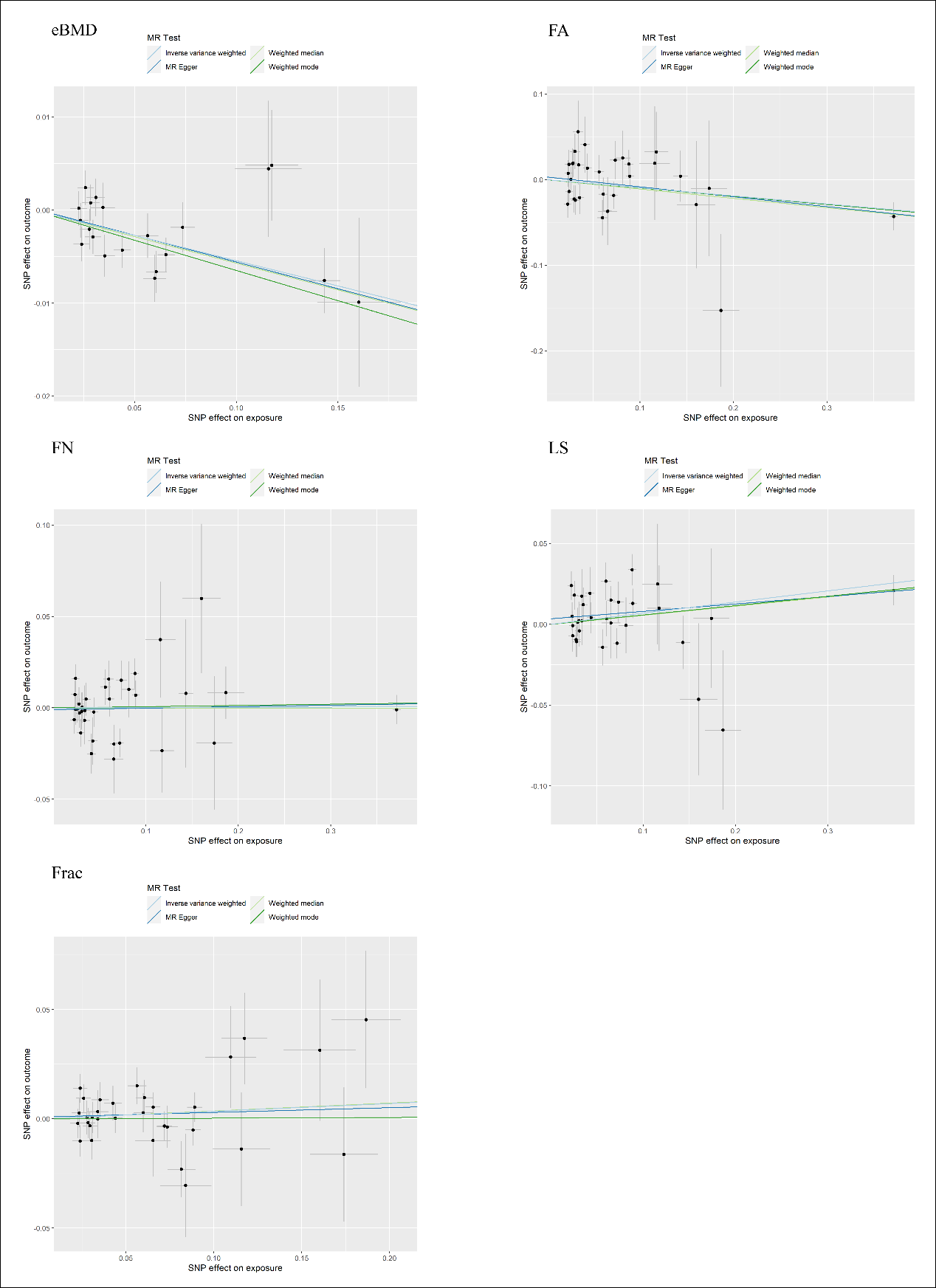


**Supplementary Figure 16.** Scatter plots of the ratio of n-6 PUFAs to n-3 PUFAs on outcomes.

**Abbreviations:** BMD: bone mineral density; eBMD: estimated BMD; FA: forearm BMD; FN: femoral neck BMD; LS: lumbar BMD; Frac: fracture.
