# Supplementary material for: Causal effect of polyunsaturated fatty acids on bone mineral density and fracture
Source: Front Nutr. 2022 Dec 8;9:1014847. doi: 10.3389/fnut.2022.1014847 (PMC9772990; doi:10.3389/fnut.2022.1014847)
Supplement: Supplementary file 2 [file Data_Sheet_2.docx]

| **Supplementary Table1.** Detailed information of studies and datasets used in the present study. | | | |
| --- | --- | --- | --- |
| **Exposure or outcome** | **PMID** | **Ancestry** | **Participants** |
| n-3 PUFAs | 35692035 | European ancestry | 114,999 individuals |
| n-6 PUFAs | 35692035 | European ancestry | 114,999 individuals |
| n-3 pct | 35692035 | European ancestry | 114,999 individuals |
| n-6 to n-3 | 35692035 | European ancestry | 114,999 individuals |
| eBMD | 30598549 | European ancestry | 426,824 individuals |
| FA BMD | 26367794 | European ancestry | 10,805 individuals |
| FN BMD | 26367794 | European ancestry | 49,988 individuals |
| LS BMD | 26367794 | European ancestry | 44,731 individuals |
| Fracture | 30598549 | European ancestry | 416,795 individuals |
| IL-6 | 27989323 | European ancestry | 8,293 individuals |
| TNF-b | 27989323 | European ancestry | 8,293 individuals |
| BMP-7 | 29875488 | European ancestry | 3,301 individuals |

**Abbreviations:** PUFAs: polyunsaturated fatty acids; n-3 pct: the ratio of n-3 fatty acids to total fatty acids; n-6 to n-3: the ratio of n-6 PUFAs to n-3 PUFAs; BMD: bone mineral density; eBMD: estimated BMD; FA: forearm; FN: femoral neck; LS: lumbar; IL: interleukin; TNF: Tumor necrosis factor; BMP: bone morphogenetic protein.

**Supplementary Table 2.** List of n-3 PUFAs genetic instruments.

| **Exposure** | **#CHR** | **POS** | **SNP** | **Effect_allele** | **Other_allele** | **Beta** | **SE** | **Pval** | **EAF** | **eBMD IVs** | **FA IVs** | **FN IVs** | **Frac IVs** | **LS IVs** |
| --- | --- | --- | --- | --- | --- | --- | --- | --- | --- | --- | --- | --- | --- | --- |
| n-3 PUFAs | 1 | 62931632 | rs1167998 | C | A | 0.071 | 0.004 | 3.60E-66 | 0.645 | rs1167998 | rs1167998 | rs1167998 | rs1167998 | rs1167998 |
| n-3 PUFAs | 1 | 109818306 | rs629301 | G | T | 0.038 | 0.005 | 1.30E-14 | 0.778 | rs633695# | rs629301 | rs629301 | rs629301 | rs629301 |
| n-3 PUFAs | 1 | 2330190 | rs6693447 | T | G | 0.023 | 0.004 | 4.80E-09 | 0.462 | rs6693447 | rs6693447 | rs6693447 | rs6693447 | rs6693447 |
| n-3 PUFAs | 2 | 21203877 | rs10184054 | C | G | -0.036 | 0.005 | 5.60E-15 | 0.224 | * | rs10184054 | rs10184054 | rs10184054 | rs10184054 |
| n-3 PUFAs | 2 | 234679384 | rs11563251 | C | T | 0.035 | 0.006 | 3.20E-08 | 0.111 | rs11563251 | rs11563251 | rs11563251 | rs11563251 | rs11563251 |
| n-3 PUFAs | 2 | 136820960 | rs11681659 | C | T | -0.025 | 0.004 | 2.00E-08 | 0.716 | rs11681248#* | rs11681659 | rs11681659 | rs11681248# | rs11681659 |
| n-3 PUFAs | 2 | 27730940 | rs1260326 | T | C | -0.082 | 0.004 | 8.40E-88 | 0.604 | rs1260326 | rs1260326 | rs6547692#* | rs1260326 | rs6547692# |
| n-3 PUFAs | 2 | 241214158 | rs13424225 | G | T | 0.022 | 0.004 | 2.20E-08 | 0.450 | rs13424225 | rs13424225 | rs13424225 | rs13424225 | rs13424225 |
| n-3 PUFAs | 2 | 20363666 | rs35135293 | C | T | -0.021 | 0.004 | 3.90E-08 | 0.517 | rs35135293 | rs35135293 | rs35135293 | rs35135293 | rs35135293 |
| n-3 PUFAs | 4 | 69491284 | rs4860987 | A | T | 0.046 | 0.005 | 1.20E-21 | 0.259 | — | rs4860987 | rs4860987 | rs4860987 | rs4860987 |
| n-3 PUFAs | 5 | 131677047 | rs11242109 | G | T | 0.024 | 0.004 | 2.40E-09 | 0.479 | rs11242109 | rs11242109 | rs11242109 | rs11242109 | rs11242109 |
| n-3 PUFAs | 5 | 156397673 | rs6882345 | G | A | 0.029 | 0.004 | 1.90E-13 | 0.633 | rs6882345 | rs6882345 | rs6882345 | rs6882345 | rs6882345 |
| n-3 PUFAs | 6 | 161010118 | rs10455872 | A | G | -0.063 | 0.008 | 2.80E-17 | 0.079 | rs10455872 | rs10455872 | rs10455872 | rs10455872 | rs10455872 |
| n-3 PUFAs | 6 | 160922870 | rs117733303 | A | G | -0.116 | 0.015 | 1.40E-15 | 0.019 | rs117733303 | — | — | rs117733303 | — |
| n-3 PUFAs | 6 | 31311912 | rs2394976 | G | T | -0.046 | 0.006 | 1.20E-15 | 0.162 | * | rs2394976 | rs2394976 | rs2394976 | rs2394976 |
| n-3 PUFAs | 6 | 32379383 | rs3129962 | G | A | -0.039 | 0.006 | 1.80E-09 | 0.130 | rs3129962 | rs3763313# | rs3763313# | rs3129962 | rs3763313 |
| n-3 PUFAs | 7 | 25990597 | rs4000713 | G | A | -0.029 | 0.004 | 1.00E-11 | 0.295 | rs4000713 | rs4000713 | rs4000713 | rs4000713 | rs4000713 |
| n-3 PUFAs | 7 | 73042085 | rs62466318 | C | T | -0.072 | 0.005 | 1.20E-45 | 0.204 | rs62466318 | rs62466318 | rs62466318 | rs62466318 | rs62466318 |
| n-3 PUFAs | 7 | 44785800 | rs73109460 | G | A | -0.035 | 0.006 | 9.20E-10 | 0.124 | rs73109459#* | rs73109460 | rs73109460 | rs73109459# | rs73109460 |
| n-3 PUFAs | 8 | 126506694 | rs112875651 | G | A | -0.087 | 0.004 | 3.50E-98 | 0.392 | rs28601761#* | rs112875651 | rs28601761#* | — | rs28601761# |
| n-3 PUFAs | 8 | 19844415 | rs7819706 | A | G | -0.040 | 0.006 | 1.80E-10 | 0.118 | * | rs7819706 | rs59147390# | rs7819706 | rs59147390# |
| n-3 PUFAs | 8 | 9183358 | rs9987289 | A | G | 0.057 | 0.007 | 3.20E-16 | 0.909 | * | rs9987289 | rs9987289 | rs9987289 | rs9987289 |
| n-3 PUFAs | 9 | 107665978 | rs1800978 | C | G | -0.037 | 0.006 | 5.20E-09 | 0.124 | rs1800978 | rs1800978 | rs2244278# | rs1800978 | rs2244278# |
| n-3 PUFAs | 10 | 96728169 | rs55891451 | A | C | 0.034 | 0.005 | 4.60E-12 | 0.202 | — | — | rs3750572# | — | — |
| n-3 PUFAs | 10 | 5247302 | rs6601924 | T | C | 0.035 | 0.006 | 8.50E-10 | 0.846 | * | — | — | rs6601924 | rs3750572 |
| n-3 PUFAs | 10 | 65191645 | rs7924036 | G | T | 0.023 | 0.004 | 5.50E-10 | 0.504 | rs7924036 | rs7924036 | rs7924036 | rs7924036 | rs7924036 |
| n-3 PUFAs | 11 | 61701898 | rs11230829 | A | G | -0.103 | 0.015 | 3.40E-12 | 0.028 | — | — | — | — | — |
| n-3 PUFAs | 11 | 61823630 | rs12226389 | T | C | -0.051 | 0.005 | 1.10E-22 | 0.186 | rs12226389 | rs12226389 | rs12226389 | rs12226389 | rs12226389 |
| n-3 PUFAs | 11 | 61453822 | rs143355652 | C | T | -0.154 | 0.020 | 9.40E-14 | 0.010 | rs143355652 | rs143355652 | rs143355652 | rs143355652 | rs143355652 |
| n-3 PUFAs | 11 | 116916060 | rs144018203 | G | C | 0.107 | 0.020 | 4.20E-08 | 0.011 | * | rs144018203 | rs144018203 | rs144018203 | rs144018203 |
| n-3 PUFAs | 11 | 61588305 | rs174564 | A | G | -0.337 | 0.004 | 1.00E-200 | 0.347 | * | rs174564 | rs174564 | * | rs174564 |
| n-3 PUFAs | 11 | 61248776 | rs3018731 | A | G | -0.035 | 0.005 | 2.00E-14 | 0.718 | — | rs3018731 | rs3018731 | rs3018731 | rs3018731 |
| n-3 PUFAs | 11 | 75450576 | rs673335 | T | C | -0.067 | 0.006 | 1.10E-34 | 0.160 | rs673335 | rs673335 | rs673335 | rs673335 | rs673335 |
| n-3 PUFAs | 11 | 116648917 | rs964184 | G | C | -0.117 | 0.006 | 8.90E-87 | 0.867 | rs964184 | rs964184 | rs964184 | rs964184 | rs964184 |
| n-3 PUFAs | 12 | 121423376 | rs7970695 | G | A | -0.025 | 0.004 | 1.20E-10 | 0.621 | * | rs7970695 | rs7970695 | rs7970695 | rs7970695 |
| n-3 PUFAs | 15 | 44027885 | rs139974673 | T | C | 0.118 | 0.013 | 2.30E-21 | 0.026 | rs139974673 | rs139974673 | rs139974673 | rs139974673 | rs139974673 |
| n-3 PUFAs | 15 | 58678720 | rs261290 | T | C | -0.114 | 0.004 | 3.90E-161 | 0.655 | rs261290 | rs261290 | rs261290 | rs261290 | rs261290 |
| n-3 PUFAs | 15 | 58569330 | rs34663616 | C | A | 0.036 | 0.006 | 4.40E-10 | 0.138 | rs34663616 | rs34663616 | rs34663616 | rs34663616 | rs34663616 |
| n-3 PUFAs | 15 | 58725839 | rs633695 | A | G | 0.084 | 0.004 | 9.10E-80 | 0.292 | * | rs633695 | rs633695 | rs633695 | rs633695 |
| n-3 PUFAs | 16 | 15501099 | rs1672811 | T | C | 0.025 | 0.005 | 3.00E-08 | 0.748 | rs1672811 | rs153001# | rs153001# | rs1672811 | rs153001# |
| n-3 PUFAs | 16 | 15127534 | rs72789541 | T | A | -0.081 | 0.004 | 5.60E-75 | 0.296 | * | rs72789541 | rs72789541 | rs72789541 | * |
| n-3 PUFAs | 17 | 44186063 | rs16940904 | C | T | -0.036 | 0.005 | 3.90E-14 | 0.227 | * | rs16940904 | rs16940904 | * | * |
| n-3 PUFAs | 18 | 47109955 | rs77960347 | A | G | 0.162 | 0.018 | 7.20E-22 | 0.013 | rs77960347 | rs149615216# | rs149615216# | rs77960347 | rs149615216# |
| n-3 PUFAs | 18 | 47158234 | rs9304381 | C | T | 0.053 | 0.005 | 5.20E-24 | 0.818 | rs9304381 | rs9304381 | rs9304381 | rs9304381 | rs9304381 |
| n-3 PUFAs | 19 | 45448036 | rs1132899 | T | C | 0.027 | 0.004 | 8.60E-11 | 0.510 | rs1132899 | rs2288912#~ | rs5157# | rs1132899 | rs5157# |
| n-3 PUFAs | 19 | 45424514 | rs157592 | A | C | 0.028 | 0.005 | 3.60E-09 | 0.185 | — | — | — | — | — |
| n-3 PUFAs | 19 | 19458388 | rs182611493 | A | G | -0.210 | 0.020 | 1.10E-27 | 0.013 | rs182611493 | rs182611493 | rs188247550# | rs182611493 | rs188247550# |
| n-3 PUFAs | 19 | 45430280 | rs5112 | C | G | 0.048 | 0.004 | 9.10E-30 | 0.534 | ~— | ~— | ~— | ~— | ~— |
| n-3 PUFAs | 19 | 19379549 | rs58542926 | C | T | -0.172 | 0.008 | 1.40E-113 | 0.074 | rs58542926 | rs739846# | rs739846# | rs58542926 | rs739846# |
| n-3 PUFAs | 19 | 11347657 | rs737338 | C | T | -0.073 | 0.011 | 3.50E-11 | 0.035 | * | rs66466742# | rs66466742# | rs737338 | rs66466742# |
| n-3 PUFAs | 20 | 39167592 | rs6129624 | G | A | -0.026 | 0.004 | 5.10E-10 | 0.335 | — | rs6129624 | rs6129624 | rs6129624 | rs6129624 |
| n-3 PUFAs | 21 | 40555561 | rs117143374 | T | C | -0.037 | 0.006 | 2.20E-10 | 0.142 | rs117143374 | rs117143374 | — | rs117143374 | — |

**Abbreviations:** PUFAs: polyunsaturated fatty acids; CHR: chromosome; POS: position; SNP: single nucleotide polymorphism; EAF: effect allele frequency; SE: standard error of beta; BMD: bone mineral density; eBMD: estimated BMD; FA: forearm; FN: femoral neck; Frac: fracture; LS: lumbar; — SNP can not be found in outcome GWAS data; ~ palindrome SNP; # proxy SNP; ~— palindrome SNP has no proxy SNPs, or proxy SNPs can not be found in GWAS data; * defined as an outlier.

**Supplementary Table 3.** List of n-6 PUFAs genetic instruments.

| **Exposure** | **#CHR** | **POS** | **SNP** | **Effect_allele** | **Other_allele** | **Beta** | **SE** | **Pval** | **EAF** | **eBMD IVs** | **FA IVs** | **FN IVs** | **Frac IVs** | **LS IVs** |
| --- | --- | --- | --- | --- | --- | --- | --- | --- | --- | --- | --- | --- | --- | --- |
| n-6 PUFAs | 1 | 62963737 | rs1002687 | G | A | 0.091 | 0.004 | 1.00E-107 | 0.645 | rs1002687 | rs1002687 | rs1002687 | rs1002687 | rs1002687 |
| n-6 PUFAs | 1 | 109817590 | rs12740374 | G | T | -0.057 | 0.005 | 1.50E-32 | 0.221 | * | rs7528419# | rs7528419# | rs12740374 | rs7528419# |
| n-6 PUFAs | 1 | 234850420 | rs199900492 | C | CA | -0.031 | 0.004 | 2.30E-15 | 0.484 | rs2587534#* | rs2587534# | rs2587534# | rs2587534# | rs2587534# |
| n-6 PUFAs | 1 | 55491853 | rs200730299 | A | C | -0.037 | 0.005 | 7.10E-12 | 0.194 | — | — | — | — | — |
| n-6 PUFAs | 1 | 25622291 | rs2986164 | G | A | -0.025 | 0.004 | 3.40E-09 | 0.536 | — | — | — | rs2986164 | — |
| n-6 PUFAs | 1 | 23784965 | rs534417 | A | G | 0.039 | 0.006 | 9.30E-11 | 0.875 | rs534417 | rs534417 | rs534417 | rs534417 | rs534417 |
| n-6 PUFAs | 2 | 27730940 | rs1260326 | T | C | -0.064 | 0.004 | 3.90E-55 | 0.604 | rs1260326 | rs1260326 | rs6547692# | rs1260326 | rs6547692# |
| n-6 PUFAs | 2 | 169828995 | rs3770586 | C | T | -0.023 | 0.004 | 7.10E-09 | 0.484 | * | rs3770586 | rs3770586 | rs3770586 | rs3770586 |
| n-6 PUFAs | 2 | 44072576 | rs4299376 | G | T | -0.035 | 0.004 | 1.10E-16 | 0.676 | rs4299376 | rs4299376 | rs4299376 | rs4299376 | rs4299376 |
| n-6 PUFAs | 2 | 21190209 | rs6547409 | C | T | -0.081 | 0.009 | 2.40E-20 | 0.051 | rs6547409 | rs6547409 | rs6547409 | rs6547409 | rs6547409 |
| n-6 PUFAs | 2 | 21319016 | rs672889 | T | G | 0.076 | 0.006 | 1.30E-41 | 0.860 | rs672889 | rs67332771# | rs34872576# | rs672889 | rs34872576# |
| n-6 PUFAs | 2 | 20369562 | rs870526 | C | T | -0.032 | 0.004 | 7.70E-16 | 0.521 | rs870526 | rs870526 | rs870526 | rs870526 | rs870526 |
| n-6 PUFAs | 4 | 3443931 | rs13108218 | A | G | -0.035 | 0.004 | 3.60E-18 | 0.615 | rs13108218 | rs13108218 | — | rs13108218 | — |
| n-6 PUFAs | 4 | 69340991 | rs4860948 | T | A | 0.028 | 0.005 | 1.90E-09 | 0.244 | * | rs4860948 | rs4860948 | rs4860948 | rs4860948 |
| n-6 PUFAs | 5 | 74635225 | rs4704210 | G | C | 0.047 | 0.004 | 6.10E-30 | 0.374 | rs3843481#* | rs4704210 | rs4704210 | rs3843481# | rs4704210 |
| n-6 PUFAs | 5 | 156397673 | rs6882345 | G | A | 0.045 | 0.004 | 1.20E-27 | 0.633 | rs6882345 | rs6882345 | rs6882345 | rs1393207~# | rs6882345 |
| n-6 PUFAs | 5 | 74472939 | rs7707394 | G | A | 0.030 | 0.004 | 1.20E-12 | 0.357 | rs7707394 | — | — | rs7707394 | — |
| n-6 PUFAs | 6 | 34729158 | rs114863007 | G | A | -0.046 | 0.007 | 7.30E-12 | 0.095 | * | rs114863007 | rs114863007 | rs114863007 | rs114863007 |
| n-6 PUFAs | 6 | 32587213 | rs28383314 | T | C | 0.039 | 0.004 | 1.70E-18 | 0.623 | rs28383314 | rs28383314 | rs28383314 | rs28383314 | rs28383314 |
| n-6 PUFAs | 6 | 32531745 | rs35603463 | T | C | 0.034 | 0.005 | 4.60E-10 | 0.567 | — | rs35603463 | rs35603463 | rs35603463 | rs35603463 |
| n-6 PUFAs | 6 | 31078836 | rs3734854 | G | A | 0.048 | 0.008 | 5.90E-11 | 0.065 | rs3734854 | rs78032704# | rs78032704# | rs3734854 | rs78032704# |
| n-6 PUFAs | 6 | 116322349 | rs6934962 | C | T | 0.023 | 0.004 | 2.20E-08 | 0.400 | rs6934962 | rs6934962 | rs6934962 | rs6934962 | rs6934962 |
| n-6 PUFAs | 6 | 160986915 | rs6938647 | A | C | -0.048 | 0.005 | 1.90E-23 | 0.782 | rs6938647 | rs6938647 | rs6938647 | rs6938647 | rs6938647 |
| n-6 PUFAs | 6 | 160400147 | rs7750288 | A | G | 0.025 | 0.004 | 1.30E-08 | 0.285 | rs7750288 | rs7750288 | rs7750288 | rs7750288 | rs7750288 |
| n-6 PUFAs | 6 | 160751531 | rs9295128 | G | T | -0.196 | 0.016 | 3.40E-36 | 0.017 | rs9295128 | rs9295128 | rs9295128 | rs9295128 | rs9295128 |
| n-6 PUFAs | 7 | 73037366 | rs55747707 | G | A | -0.049 | 0.005 | 1.70E-22 | 0.204 | rs55747707 | rs55747707 | rs55747707 | rs55747707 | rs55747707 |
| n-6 PUFAs | 8 | 126506694 | rs112875651 | G | A | -0.064 | 0.004 | 2.20E-53 | 0.392 | rs28601761#* | rs112875651 | rs112875651 | rs112875651 | rs112875651 |
| n-6 PUFAs | 8 | 9187242 | rs1461729 | A | G | 0.084 | 0.007 | 2.80E-36 | 0.899 | * | rs1461729 | rs1461729 | rs1461729 | rs1461729 |
| n-6 PUFAs | 8 | 116658583 | rs2737245 | G | T | -0.027 | 0.005 | 1.40E-09 | 0.279 | * | rs2737245 | rs2737245 | rs2737245 | rs2737245 |
| n-6 PUFAs | 8 | 59377357 | rs6471717 | G | A | -0.029 | 0.004 | 4.00E-12 | 0.663 | * | rs6471717 | rs6471717 | rs6471717 | rs6471717 |
| n-6 PUFAs | 8 | 141633257 | rs7831074 | C | G | 0.028 | 0.005 | 4.60E-08 | 0.759 | — | — | — | — | — |
| n-6 PUFAs | 9 | 136149711 | rs115478735 | A | T | 0.042 | 0.005 | 2.20E-17 | 0.183 | — | — | — | — | — |
| n-6 PUFAs | 9 | 107647019 | rs11789603 | C | T | 0.048 | 0.006 | 9.70E-14 | 0.109 | * | — | — | rs11789603 | — |
| n-6 PUFAs | 9 | 107661742 | rs2740488 | A | C | -0.050 | 0.005 | 5.40E-28 | 0.265 | rs2740488 | rs2740488 | rs2740488 | rs2740488 | rs2740488 |
| n-6 PUFAs | 9 | 15300968 | rs4008004 | C | A | 0.033 | 0.005 | 8.40E-12 | 0.222 | rs4008004 | rs10810374# | rs10810374# | rs4008004 | rs10810374# |
| n-6 PUFAs | 10 | 45998984 | rs148063610 | CAAATAAAT | C | -0.032 | 0.005 | 4.00E-11 | 0.763 | — | — | — | — | — |
| n-6 PUFAs | 10 | 5257647 | rs75406471 | G | A | -0.031 | 0.006 | 2.70E-08 | 0.155 | * | rs75406471 | rs75406471 | rs75406471 | rs75406471 |
| n-6 PUFAs | 11 | 116714293 | rs141469619 | A | G | 0.111 | 0.021 | 1.40E-08 | 0.010 | rs141469619 | rs141469619 | rs141469619 | rs141469619 | rs141469619 |
| n-6 PUFAs | 11 | 116817978 | rs200671503 | TTA | T | 0.051 | 0.009 | 2.10E-09 | 0.942 | — | — | — | — | — |
| n-6 PUFAs | 11 | 47643891 | rs3817335 | T | A | -0.028 | 0.004 | 9.80E-12 | 0.351 | * | rs3817335 | rs3817335 | rs3817335 | rs3817335 |
| n-6 PUFAs | 11 | 75474195 | rs72997616 | C | A | -0.052 | 0.007 | 1.60E-13 | 0.094 | rs72997616 | rs72997616 | rs72997616 | rs72997616 | rs72997616 |
| n-6 PUFAs | 11 | 116648917 | rs964184 | G | C | -0.139 | 0.006 | 1.10E-125 | 0.867 | * | rs964184 | rs964184 | rs964184 | rs964184 |
| n-6 PUFAs | 12 | 111904371 | rs4766578 | T | A | 0.028 | 0.004 | 1.50E-12 | 0.503 | rs3184504~#* | rs3184504~# | rs7310615~#* | rs10774625~# | rs10774625~# |
| n-6 PUFAs | 12 | 121415293 | rs7139079 | G | A | -0.030 | 0.004 | 3.30E-13 | 0.593 | rs7139079 | rs7139079 | rs7139079 | rs7139079 | rs7139079 |
| n-6 PUFAs | 13 | 114547372 | rs6602911 | C | T | 0.026 | 0.004 | 1.30E-09 | 0.360 | rs6602911 | rs6602911 | rs6602911 | rs6602911 | rs6602911 |
| n-6 PUFAs | 15 | 58678720 | rs261290 | T | C | -0.097 | 0.004 | 1.00E-116 | 0.655 | * | rs261290 | rs261290 | rs261290 | rs261290 |
| n-6 PUFAs | 15 | 58725839 | rs633695 | A | G | 0.073 | 0.004 | 1.30E-59 | 0.292 | * | rs633695 | rs633695 | rs633695 | rs633695 |
| n-6 PUFAs | 16 | 56991363 | rs183130 | C | T | 0.062 | 0.004 | 1.40E-48 | 0.324 | rs183130* | rs247617# | rs247617# | rs183130 | rs247617# |
| n-6 PUFAs | 17 | 45781599 | rs4439799 | C | T | 0.022 | 0.004 | 1.30E-08 | 0.502 | rs4439799 | rs4439799 | rs4439799 | rs4439799 | rs4439799 |
| n-6 PUFAs | 17 | 67082962 | rs740516 | C | G | -0.032 | 0.006 | 1.40E-08 | 0.151 | rs740516 | — | — | rs740516 | — |
| n-6 PUFAs | 18 | 47109955 | rs77960347 | A | G | 0.276 | 0.018 | 2.80E-56 | 0.013 | rs77960347* | rs149615216# | rs149615216# | rs77960347 | rs149615216# |
| n-6 PUFAs | 18 | 47158234 | rs9304381 | C | T | 0.070 | 0.005 | 7.20E-42 | 0.818 | rs9304381 | rs9304381 | rs9304381 | rs9304381 | rs9304381 |
| n-6 PUFAs | 19 | 45413233 | rs1065853 | G | T | -0.199 | 0.007 | 3.60E-160 | 0.081 | — | — | — | — | — |
| n-6 PUFAs | 19 | 45412955 | rs1081105 | A | C | 0.119 | 0.012 | 1.80E-22 | 0.028 | rs1081105 | rs1081105 | rs1081105 | rs1081105 | rs1081105 |
| n-6 PUFAs | 19 | 11190534 | rs142158911 | G | A | -0.094 | 0.006 | 5.20E-52 | 0.117 | rs142158911 | rs142158911 | rs142158911 | rs142158911 | rs142158911 |
| n-6 PUFAs | 19 | 11346155 | rs56322906 | G | A | -0.100 | 0.011 | 1.20E-19 | 0.035 | * | rs66466742# | rs66466742# | rs56322906 | rs66466742# |
| n-6 PUFAs | 19 | 19379549 | rs58542926 | C | T | -0.128 | 0.008 | 2.50E-65 | 0.074 | rs58542926 | rs739846# | rs739846# | rs58542926 | rs739846# |
| n-6 PUFAs | 19 | 45445517 | rs79429216 | G | A | 0.151 | 0.018 | 1.30E-17 | 0.013 | rs79429216 | — | — | rs79429216 | — |
| n-6 PUFAs | 20 | 43042364 | rs1800961 | C | T | -0.074 | 0.012 | 3.30E-10 | 0.030 | rs1800961 | — | — | rs1800961 | — |
| n-6 PUFAs | 20 | 39179822 | rs1883711 | G | C | 0.092 | 0.012 | 3.20E-16 | 0.031 | rs1883711 | rs1883711 | rs1883711 | rs1883711 | rs1883711 |
| n-6 PUFAs | 20 | 34150207 | rs2378390 | G | A | -0.033 | 0.006 | 3.20E-09 | 0.141 | — | — | — | — | — |
| n-6 PUFAs | 22 | 21916272 | rs5754102 | C | A | -0.032 | 0.005 | 9.90E-10 | 0.183 | rs5754100#* | rs5754102 | rs5754102 | [rs5754100#](https://pubs.broadinstitute.org/mammals/haploreg/detail_v2.php?query=&id=rs5754100) | rs5754102 |
| n-6 PUFAs | 22 | 50868669 | rs9616847 | A | T | 0.024 | 0.004 | 1.40E-08 | 0.388 | * | rs9616847 | rs9616847 | rs9616847 | rs9616847 |

**Abbreviations:** PUFAs: polyunsaturated fatty acids; CHR: chromosome; POS: position; SNP: single nucleotide polymorphism; EAF: effect allele frequency; SE: standard error of beta; BMD: bone mineral density; eBMD: estimated BMD; FA: forearm; FN: femoral neck; Frac: fracture; LS: lumbar; — SNP can not be found in outcome GWAS data; ~ palindrome SNP; # proxy SNP; ~— palindrome SNP has no proxy SNPs, or proxy SNPs can not be found in GWAS data; * defined as an outlier.

**Supplementary Table 4.** List of the ratio of n-3 fatty acids to total fatty acids genetic instruments.

| **Exposure** | **#CHR** | **POS** | **SNP** | **Effect_allele** | **Other_allele** | **Beta** | **SE** | **Pval** | **EAF** | **eBMD IVs** | **FA IVs** | **FN IVs** | **Frac IVs** | **LS IVs** |
| --- | --- | --- | --- | --- | --- | --- | --- | --- | --- | --- | --- | --- | --- | --- |
| n-3 pct | 1 | 62906489 | rs638714 | G | T | -0.033 | 0.004 | 4.90E-15 | 0.346 | rs638714 | rs638714 | rs638714 | rs638714 | rs638714 |
| n-3 pct | 1 | 2330190 | rs6693447 | T | G | 0.026 | 0.004 | 8.30E-12 | 0.462 | rs6693447 | rs6693447 | rs6693447 | rs6693447 | rs6693447 |
| n-3 pct | 2 | 27730940 | rs1260326 | T | C | -0.038 | 0.004 | 2.30E-19 | 0.604 | * | rs1260326 | rs6547692# | rs1260326 | rs6547692# |
| n-3 pct | 2 | 136817616 | rs2011946 | C | A | -0.027 | 0.005 | 2.60E-09 | 0.735 | * | rs2011946 | rs2011946 | rs2011946 | rs2011946 |
| n-3 pct | 4 | 69491284 | rs4860987 | A | T | 0.041 | 0.005 | 8.10E-17 | 0.259 | — | rs4860987 | rs4860987 | rs4860987 | rs4860987 |
| n-3 pct | 5 | 131665423 | rs272888 | T | C | 0.029 | 0.004 | 1.80E-11 | 0.707 | rs272888 | rs272888 | rs272888 | rs272888 | rs272888 |
| n-3 pct | 5 | 87730027 | rs7444298 | A | G | 0.027 | 0.005 | 2.40E-08 | 0.237 | rs7444298 | rs7444298 | rs7444298 | rs7444298 | rs7444298 |
| n-3 pct | 6 | 31311912 | rs2394976 | G | T | -0.031 | 0.006 | 5.40E-09 | 0.162 | * | rs2394976 | rs2394976 | rs2394976 | rs2394976 |
| n-3 pct | 6 | 160564476 | rs662138 | C | G | -0.034 | 0.005 | 6.60E-11 | 0.186 | * | rs662138 | — | rs662138 | — |
| n-3 pct | 7 | 25990597 | rs4000713 | G | A | -0.027 | 0.004 | 1.60E-10 | 0.295 | rs4000713 | rs4000713 | rs4000713 | rs4000713 | rs4000713 |
| n-3 pct | 7 | 73042085 | rs62466318 | C | T | -0.041 | 0.005 | 3.10E-16 | 0.204 | * | rs62466318 | rs62466318 | rs62466318 | rs62466318 |
| n-3 pct | 7 | 44785800 | rs73109460 | G | A | -0.032 | 0.006 | 7.80E-09 | 0.124 | rs73109459# | rs73109460 | rs73109460 | rs73109459# | rs73109460 |
| n-3 pct | 8 | 126506694 | rs112875651 | G | A | -0.052 | 0.004 | 5.70E-35 | 0.392 | rs28601761#* | rs112875651 | rs112875651 | rs112875651 | rs28601761~# |
| n-3 pct | 9 | 139572068 | rs2236514 | C | G | -0.022 | 0.004 | 3.10E-08 | 0.658 | * | rs2236514 | rs9411262# | rs9411262~# | rs9411262~# |
| n-3 pct | 10 | 96717286 | rs56233220 | G | C | 0.036 | 0.005 | 4.40E-13 | 0.200 | rs4086116#* | rs56233220 | rs56233220 | rs61886778# | rs56233220 |
| n-3 pct | 10 | 65191645 | rs7924036 | G | T | 0.038 | 0.004 | 4.00E-23 | 0.504 | rs7924036 | rs7924036 | rs7924036 | rs7924036 | rs7924036 |
| n-3 pct | 11 | 61701898 | rs11230829 | A | G | -0.116 | 0.015 | 1.70E-15 | 0.028 | — | — | — | — | — |
| n-3 pct | 11 | 75449819 | rs11236512 | C | A | -0.071 | 0.009 | 5.30E-15 | 0.063 | — | — | — | — | — |
| n-3 pct | 11 | 61823630 | rs12226389 | T | C | -0.062 | 0.005 | 2.20E-32 | 0.186 | rs12226389 | rs12226389 | rs12226389 | rs12226389 | rs12226389 |
| n-3 pct | 11 | 61453822 | rs143355652 | C | T | -0.179 | 0.020 | 1.20E-18 | 0.010 | rs143355652 | rs143355652 | rs143355652 | rs143355652 | rs143355652 |
| n-3 pct | 11 | 61406089 | rs145786300 | G | A | -0.187 | 0.019 | 1.30E-22 | 0.012 | * | rs145786300 | rs145786300 | rs145786300 | rs145786300 |
| n-3 pct | 11 | 68525539 | rs149402055 | C | T | -0.173 | 0.015 | 9.20E-30 | 0.020 | rs149402055 | rs149402055 | rs149402055 | rs149402055 | rs149402055 |
| n-3 pct | 11 | 61588305 | rs174564 | A | G | -0.392 | 0.004 | 1.00E-200 | 0.347 | * | rs174564 | rs174564 | rs174564 | rs174564 |
| n-3 pct | 11 | 61844663 | rs191623731 | T | G | 0.118 | 0.016 | 9.80E-14 | 0.016 | rs191623731 | rs191623731 | rs191623731 | rs191623731 | rs191623731 |
| n-3 pct | 11 | 60899701 | rs2232143 | T | C | 0.108 | 0.014 | 1.30E-13 | 0.022 | * | — | — | rs2232143 | — |
| n-3 pct | 11 | 75453974 | rs75227397 | G | A | -0.063 | 0.012 | 2.80E-08 | 0.031 | rs75227397 | rs75227397 | rs75227397 | rs75227397 | rs75227397 |
| n-3 pct | 11 | 116648917 | rs964184 | G | C | -0.038 | 0.006 | 1.20E-10 | 0.867 | rs964184 | rs964184 | rs964184 | rs964184 | rs964184 |
| n-3 pct | 13 | 56069705 | rs9563335 | A | G | -0.044 | 0.008 | 5.50E-09 | 0.862 | — | rs9563335 | rs9563335 | rs9563335 | rs9563335 |
| n-3 pct | 15 | 58724706 | rs11632618 | G | A | 0.078 | 0.008 | 1.10E-22 | 0.070 | rs11632618 | rs11632618 | rs11632618 | rs11632618 | rs11632618 |
| n-3 pct | 15 | 44027885 | rs139974673 | T | C | 0.084 | 0.013 | 1.30E-11 | 0.026 | rs139974673 | rs139974673 | rs139974673 | rs139974673 | rs139974673 |
| n-3 pct | 15 | 58580781 | rs1560390 | T | C | -0.032 | 0.005 | 6.90E-12 | 0.220 | rs1560390 | rs1560390 | rs1560390 | rs1560390 | rs1560390 |
| n-3 pct | 15 | 58680178 | rs261291 | T | C | 0.081 | 0.004 | 1.80E-83 | 0.356 | rs261291 | rs261291 | rs261291 | rs261291 | rs261291 |
| n-3 pct | 16 | 15678414 | rs35390787 | CA | C | -0.027 | 0.004 | 5.60E-11 | 0.562 | — | — | — | — | — |
| n-3 pct | 16 | 15127534 | rs72789541 | T | A | -0.097 | 0.004 | 3.50E-106 | 0.296 | * | rs72789541 | rs72789541 | rs72789542# | rs72789541 |
| n-3 pct | 17 | 44186063 | rs16940904 | C | T | -0.042 | 0.005 | 2.40E-19 | 0.227 | * | rs16940904 | rs16940904 | rs16940904 | rs16940904 |
| n-3 pct | 17 | 17407191 | rs8074191 | T | C | -0.027 | 0.005 | 2.30E-08 | 0.756 | rs8074191 | rs8074191 | rs8074191 | rs8074191 | rs8074191 |
| n-3 pct | 18 | 47166694 | rs9947684 | A | G | 0.029 | 0.004 | 2.80E-12 | 0.654 | rs9947684 | rs9947684 | rs9947684 | rs9947684 | rs9947684 |
| n-3 pct | 19 | 19458388 | rs182611493 | A | G | -0.172 | 0.020 | 8.90E-19 | 0.013 | rs182611493 | rs182611493 | rs188247550# | rs182611493 | rs188247550# |
| n-3 pct | 19 | 45441475 | rs190921611 | G | A | 0.032 | 0.005 | 2.00E-11 | 0.311 | — | rs190921611 | rs190921611 | — | rs190921611 |
| n-3 pct | 19 | 19379549 | rs58542926 | C | T | -0.131 | 0.008 | 1.20E-67 | 0.074 | rs58542926 | rs739846# | rs739846# | rs58542926 | rs739846# |
| n-3 pct | 19 | 45414399 | rs72654473 | C | A | 0.037 | 0.007 | 6.10E-09 | 0.108 | * | rs72654473 | rs72654473 | rs72654473 | rs72654473 |

**Abbreviations:** PUFAs: polyunsaturated fatty acids; CHR: chromosome; POS: position; SNP: single nucleotide polymorphism; EAF: effect allele frequency; SE: standard error of beta; BMD: bone mineral density; eBMD: estimated BMD; FA: forearm; FN: femoral neck; Frac: fracture; LS: lumbar; — SNP can not be found in outcome GWAS data; ~ palindrome SNP; # proxy SNP; ~— palindrome SNP has no proxy SNPs, or proxy SNPs can not be found in GWAS data; * defined as an outlier.

**Supplementary Table 5.** List of the ratio of n-6 PUFAs to n-3 PUFAs genetic instruments.

| **Exposure** | **#CHR** | **POS** | **SNP** | **Effect_allele** | **Other_allele** | **Beta** | **SE** | **Pval** | **EAF** | **eBMD IVs** | **FA IVs** | **FN IVs** | **Frac IVs** | **LS IVs** |
| --- | --- | --- | --- | --- | --- | --- | --- | --- | --- | --- | --- | --- | --- | --- |
| n6 to n3 | 1 | 62906489 | rs638714 | G | T | 0.044 | 0.004 | 3.70E-25 | 0.346 | rs638714 | rs638714 | rs638714 | rs638714 | rs638714 |
| n6 to n3 | 1 | 2329661 | rs6698680 | A | G | -0.026 | 0.004 | 2.10E-11 | 0.462 | rs6698680 | rs6698680 | rs6698680 | rs6698680 | rs6698680 |
| n6 to n3 | 2 | 27730940 | rs1260326 | T | C | 0.065 | 0.004 | 4.20E-55 | 0.604 | rs1260326 | rs1260326 | rs6547692# | rs1260326 | rs6547692# |
| n6 to n3 | 4 | 69491284 | rs4860987 | A | T | -0.043 | 0.005 | 6.80E-18 | 0.259 | — | — | rs4860987# | rs4860987 | rs4860987 |
| n6 to n3 | 5 | 131677047 | rs11242109 | G | T | -0.024 | 0.004 | 1.40E-09 | 0.479 | rs11242109 | rs11242109 | rs11242109 | rs11242109 | rs11242109 |
| n6 to n3 | 6 | 160578860 | rs1564348 | T | C | 0.030 | 0.005 | 1.40E-08 | 0.170 | * | rs1564348 | rs1564348 | rs1564348 | rs1564348 |
| n6 to n3 | 6 | 31311912 | rs2394976 | G | T | 0.034 | 0.006 | 5.80E-10 | 0.162 | * | rs2394976 | rs2394976 | rs2394976 | rs2394976 |
| n6 to n3 | 7 | 25990597 | rs4000713 | G | A | 0.031 | 0.004 | 6.10E-13 | 0.295 | rs4000713 | rs4000713 | rs4000713 | rs4000713 | rs4000713 |
| n6 to n3 | 7 | 73042085 | rs62466318 | C | T | 0.061 | 0.005 | 4.40E-33 | 0.204 | rs62466318 | rs62466318 | rs62466318 | rs62466318 | rs62466318 |
| n6 to n3 | 7 | 44785800 | rs73109460 | G | A | 0.034 | 0.006 | 2.20E-09 | 0.124 | — | — | rs73109460 | [rs73109459#](https://pubs.broadinstitute.org/mammals/haploreg/detail_v2.php?query=&id=rs73109459) | rs73109460 |
| n6 to n3 | 8 | 126506694 | rs112875651 | G | A | 0.072 | 0.004 | 6.50E-65 | 0.392 | * | rs28601761~# | rs112875651 | rs112875651 | rs112875651 |
| n6 to n3 | 8 | 19940058 | rs13273454 | C | T | 0.023 | 0.004 | 5.80E-09 | 0.471 | rs13273454 | rs13273454 | rs13273454 | rs13273454 | rs13273454 |
| n6 to n3 | 9 | 16048248 | rs10733306 | C | T | 0.023 | 0.004 | 3.20E-08 | 0.460 | rs10733306 | rs10733306 | rs10733306 | rs10733306 | rs10733306 |
| n6 to n3 | 10 | 96728169 | rs55891451 | A | C | -0.036 | 0.005 | 1.20E-12 | 0.202 | — | — | — | — | — |
| n6 to n3 | 10 | 64988931 | rs7916868 | A | T | -0.030 | 0.004 | 5.10E-14 | 0.504 | rs10822149~# | rs10822149~# | rs10822149~# | rs7916868~# | rs10822149~# |
| n6 to n3 | 11 | 61701898 | rs11230829 | A | G | 0.102 | 0.015 | 4.00E-12 | 0.028 | — | — | — | — | — |
| n6 to n3 | 11 | 61823630 | rs12226389 | T | C | 0.056 | 0.005 | 8.40E-27 | 0.186 | rs12226389 | rs12226389 | rs12226389 | rs12226389 | rs12226389 |
| n6 to n3 | 11 | 61453822 | rs143355652 | C | T | 0.160 | 0.021 | 7.60E-15 | 0.010 | rs143355652 | rs143355652 | rs143355652 | rs143355652 | rs143355652 |
| n6 to n3 | 11 | 61850279 | rs145659493 | C | A | -0.116 | 0.016 | 4.30E-13 | 0.016 | rs145659493 | rs145659493 | rs145659493 | rs145659493 | rs145659493 |
| n6 to n3 | 11 | 61406089 | rs145786300 | G | A | 0.174 | 0.019 | 1.10E-19 | 0.012 | * | rs145786300 | rs145786300 | rs145786300 | rs145786300 |
| n6 to n3 | 11 | 61983775 | rs149820547 | T | G | 0.065 | 0.010 | 1.20E-10 | 0.042 | * | rs149820547 | rs149820547 | rs149820547 | rs149820547 |
| n6 to n3 | 11 | 61588305 | rs174564 | A | G | 0.371 | 0.004 | 1.00E-200 | 0.347 | * | rs174564 | rs174564 | * | rs174564 |
| n6 to n3 | 11 | 60899701 | rs2232143 | T | C | -0.110 | 0.015 | 9.40E-14 | 0.022 | * | — | — | rs2232143 | — |
| n6 to n3 | 11 | 75450576 | rs673335 | T | C | 0.060 | 0.006 | 7.40E-28 | 0.160 | rs673335 | rs673335 | rs673335 | rs673335 | rs673335 |
| n6 to n3 | 11 | 116648917 | rs964184 | G | C | 0.074 | 0.006 | 5.30E-35 | 0.867 | rs964184 | rs964184 | rs964184 | rs964184 | rs964184 |
| n6 to n3 | 15 | 58724706 | rs11632618 | G | A | -0.082 | 0.008 | 1.20E-24 | 0.070 | * | rs11632618 | rs11632618 | rs11632618 | rs11632618 |
| n6 to n3 | 15 | 44027885 | rs139974673 | T | C | -0.117 | 0.013 | 9.10E-21 | 0.026 | rs139974673 | rs139974673 | rs139974673 | rs139974673 | rs139974673 |
| n6 to n3 | 15 | 58580781 | rs1560390 | T | C | 0.035 | 0.005 | 2.80E-13 | 0.220 | rs1560390 | rs1560390 | rs1560390 | rs1560390 | rs1560390 |
| n6 to n3 | 15 | 58680178 | rs261291 | T | C | -0.089 | 0.004 | 9.90E-99 | 0.356 | * | rs261291 | rs261291 | rs261291 | rs261291 |
| n6 to n3 | 16 | 15678414 | rs35390787 | CA | C | 0.025 | 0.004 | 9.70E-10 | 0.562 | — | — | — | — | — |
| n6 to n3 | 16 | 15127534 | rs72789541 | T | A | 0.088 | 0.004 | 1.40E-86 | 0.296 | * | rs72789541 | rs72789541 | rs72789541 | rs72789541 |
| n6 to n3 | 17 | 44186063 | rs16940904 | C | T | 0.041 | 0.005 | 2.30E-18 | 0.227 | * | rs16940904 | rs16940904 | * | * |
| n6 to n3 | 17 | 73888423 | rs7222755 | A | G | 0.024 | 0.005 | 4.10E-08 | 0.291 | * | rs7222755 | rs7207180# | rs7222755 | rs7207180# |
| n6 to n3 | 17 | 17407191 | rs8074191 | T | C | 0.028 | 0.005 | 4.80E-09 | 0.756 | rs8074191 | rs8074191 | rs8074191 | rs8074191 | rs8074191 |
| n6 to n3 | 18 | 47166694 | rs9947684 | A | G | -0.028 | 0.004 | 5.70E-12 | 0.654 | rs9947684 | rs9947684 | rs9947684 | rs9947684 | rs9947684 |
| n6 to n3 | 19 | 45413233 | rs1065853 | G | T | -0.079 | 0.008 | 1.20E-26 | 0.081 | — | — | — | — | — |
| n6 to n3 | 19 | 8429323 | rs116843064 | G | A | 0.084 | 0.015 | 1.60E-08 | 0.020 | * | — | — | rs116843064 | — |
| n6 to n3 | 19 | 45441907 | rs12976395 | G | C | -0.027 | 0.004 | 7.50E-10 | 0.505 | — | — | ~— | — | ~— |
| n6 to n3 | 19 | 19458388 | rs182611493 | A | G | 0.187 | 0.020 | 1.10E-21 | 0.013 | * | rs182611493 | rs188247550# | rs182611493 | rs188247550# |
| n6 to n3 | 19 | 19379549 | rs58542926 | C | T | 0.143 | 0.008 | 3.40E-79 | 0.074 | rs58542926 | rs73001065# | rs739846# | * | rs73984#6 |
| n6 to n3 | 21 | 40555561 | rs117143374 | T | C | 0.034 | 0.006 | 6.00E-09 | 0.142 | rs117143374 | rs117143374 | — | * | — |

**Abbreviations:** PUFAs: polyunsaturated fatty acids; CHR: chromosome; POS: position; SNP: single nucleotide polymorphism; EAF: effect allele frequency; SE: standard error of beta; BMD: bone mineral density; eBMD: estimated BMD; FA: forearm; FN: femoral neck; Frac: fracture; LS: lumbar; — SNP can not be found in outcome GWAS data; ~ palindrome SNP; # proxy SNP; ~— palindrome SNP has no proxy SNPs, or proxy SNPs can not be found in GWAS data; * defined as an outlier.

**Supplementary Table 6.** List of the circulating level of cytokines genetic instruments on PUFAs.

| **Exposure** | **#CHR** | **POS** | **SNP** | **Effect_allele** | **Other_allele** | **Beta** | **SE** | **Pval** | **EAF** | **n-3 PUFAs IVs** | **n-3 pct Ivs** | **n-6 PUFAs Ivs** | **n-6 to n-3 Ivs** |
| --- | --- | --- | --- | --- | --- | --- | --- | --- | --- | --- | --- | --- | --- |
| IL-6^#^ | 2 | 224874874 | rs13412535 | G | A | 0.116 | 0.022 | 7.34E-08 | 0.220 | rs13412535 | rs13412535 | rs13412535 | rs13412535 |
| IL-6^#^ | 17 | 45722293 | rs72831623 | A | G | 0.197 | 0.037 | 1.08E-07 | 0.060 | rs72831623 | rs72831623 | rs72831623 | rs72831623 |
| TNF-b | 1 | 22821844 | rs78296352 | T | G | 1.222 | 0.137 | 4.76E-21 | 0.030 | rs78296352 | rs78296352 | rs78296352 | rs78296352 |
| TNF-b | 1 | 23047050 | rs116196280 | T | G | 0.718 | 0.101 | 4.98E-13 | 0.020 | rs116196280 | rs116196280 | rs116196280 | rs116196280 |
| BMP-7 | 4 | 187153786 | rs4241818 | C | T | 0.207 | 0.025 | 3.72E-17 | 0.514 | rs4241818 | rs4241818 | rs4241818 | rs4241818 |
| BMP-7 | 5 | 176839890 | rs2731674 | G | T | 0.162 | 0.028 | 8.71E-09 | 0.753 | rs2731674 | rs2731674 | rs2731674 | rs2731674 |

**Abbreviations:** CHR: chromosome; POS: position; SNP: single nucleotide polymorphism; EAF: effect allele frequency; SE: standard error of beta; UFAs: polyunsaturated fatty acids; n-3 pct: the ratio of n-3 fatty acids to total fatty acids; n-6 to n-3: the ratio of n-6 PUFAs to n-3 PUFAs; IL: interleukin; TNF: Tumor necrosis factor; BMP: bone morphogenetic protein; ^#^ No genetic variants were available on p< 5×10^-08^ threshold. Thus, genetic variants were identified using a more liberal threshold of p< 5×10^-07^.

**Supplementary Table 7.** List of the circulating level of cytokines genetic instruments on BMD.

| **Exposure** | **#CHR** | **POS** | **SNP** | **Effect_allele** | **Other_allele** | **Beta** | **SE** | **Pval** | **EAF** | **eBMD IVs** | **FA IVs** | **FN IVs** | **Frac IVs** | **LS IVs** |
| --- | --- | --- | --- | --- | --- | --- | --- | --- | --- | --- | --- | --- | --- | --- |
| IL-6^#^ | 2 | 224874874 | rs13412535 | G | A | 0.116 | 0.022 | 7.34E-08 | 0.220 | rs13412535 | rs13412535 | rs13412535 | rs13412535 | rs13412535 |
| IL-6^#^ | 17 | 45722293 | rs72831623 | A | G | 0.197 | 0.037 | 1.08E-07 | 0.060 | — | rs72831623 | rs72831623 | rs72831623 | rs72831623 |
| TNF-b | 1 | 22821844 | rs78296352 | T | G | 1.222 | 0.137 | 4.76E-21 | 0.030 | rs78296352 | rs78296352 | rs78296352 | rs78296352 | rs78296352 |
| TNF-b | 1 | 23047050 | rs116196280 | T | G | 0.718 | 0.101 | 4.98E-13 | 0.020 | rs116196280 | rs116196280 | rs116196280 | rs116196280 | rs116196280 |
| BMP-7 | 4 | 187153786 | rs4241818 | C | T | 0.207 | 0.025 | 3.72E-17 | 0.514 | rs4241818 | rs4241818 | rs4241818 | rs4241818 | rs4241818 |
| BMP-7 | 5 | 176839890 | rs2731674 | G | T | 0.162 | 0.028 | 8.71E-09 | 0.753 | rs2731674 | rs2731674 | rs2731674 | rs2731674 | rs2731674 |

**Abbreviations:** CHR: chromosome; POS: position; SNP: single nucleotide polymorphism; EAF: effect allele frequency; SE: standard error of beta; BMD: bone mineral density; eBMD: estimated BMD; FA: forearm; FN: femoral neck; Frac: fracture; LS: lumbar; IL: interleukin; TNF: Tumor necrosis factor; BMP: bone morphogenetic protein;— SNP can not be found in outcome GWAS data and has no proxy SNPs, or proxy SNPs can not be found in outcome GWAS data; ^#^ No genetic variants were available on p< 5×10^-08^ threshold. Thus, genetic variants were identified using a more liberal threshold of p< 5×10^-07^.

**Supplementary Table 8.** MR estimate results of n-3 PUFAs on outcomes.

| **Outcome** | | **Methods** | **Nsnp** | **Beta** | **SE** | ***P* value** | **OR** | **or_lci95** | **or_uci95** | **Horizontal pleiotropy** | | | | **Heterogeneity** | |
| --- | --- | --- | --- | --- | --- | --- | --- | --- | --- | --- | --- | --- | --- | --- | --- |
|  |  |  |  |  |  |  |  |  |  | **MR-Egger regression** | | | **MR-PRESSO** | **Cochran’s *Q*** | ***P* value** |
|  |  |  |  |  |  |  |  |  |  | **Egger intercept** | **SE** | ***P* value** | **Global test *P* value** |  |  |
| eBMD | Weighted median | | 30 | 0.021 | 0.012 | 0.073 | 1.021 | 0.998 | 1.045 | 0.001 | 0.001 | 0.410 | 0.050 | 42.136 | 0.055 |
|  | MR Egger | | 30 | 0.019 | 0.015 | 0.219 | 1.019 | 0.989 | 1.050 |  |  |  |  |  |  |
|  | Weighted mode | | 30 | 0.020 | 0.013 | 0.129 | 1.021 | 0.995 | 1.047 |  |  |  |  |  |  |
|  | IVW | | 30 | 0.030 | 0.009 | 0.001* | 1.030 | 1.013 | 1.047 |  |  |  |  |  |  |
| FA | Weighted median | | 45 | 0.100 | 0.047 | 0.034 | 1.105 | 1.008 | 1.212 | 0.001 | 0.005 | 0.787 | 0.260 | 50.464 | 0.233 |
|  | MR Egger | | 45 | 0.076 | 0.055 | 0.171 | 1.079 | 0.969 | 1.201 |  |  |  |  |  |  |
|  | Weighted mode | | 45 | 0.075 | 0.045 | 0.101 | 1.078 | 0.987 | 1.176 |  |  |  |  |  |  |
|  | IVW | | 45 | 0.086 | 0.038 | 0.025* | 1.090 | 1.011 | 1.176 |  |  |  |  |  |  |
| FN | Weighted median | | 44 | 0.001 | 0.023 | 0.973 | 1.001 | 0.957 | 1.046 | 0.001 | 0.002 | 0.599 | 0.162 | 54.523 | 0.112 |
|  | MR Egger | | 44 | -0.022 | 0.028 | 0.435 | 0.978 | 0.927 | 1.033 |  |  |  |  |  |  |
|  | Weighted mode | | 44 | -0.004 | 0.022 | 0.873 | 0.996 | 0.954 | 1.040 |  |  |  |  |  |  |
|  | IVW | | 44 | -0.012 | 0.020 | 0.557 | 0.988 | 0.950 | 1.028 |  |  |  |  |  |  |
| Frac | Weighted median | | 45 | -0.033 | 0.035 | 0.356 | 0.968 | 0.903 | 1.037 | <0.001 | 0.002 | 0.934 | 0.368 | 48.104 | 0.310 |
|  | MR Egger | | 45 | -0.022 | 0.044 | 0.627 | 0.979 | 0.898 | 1.067 |  |  |  |  |  |  |
|  | Weighted mode | | 45 | -0.004 | 0.038 | 0.921 | 0.996 | 0.924 | 1.074 |  |  |  |  |  |  |
|  | IVW | | 45 | -0.018 | 0.023 | 0.433 | 0.982 | 0.938 | 1.028 |  |  |  |  |  |  |
| LS | Weighted median | | 44 | 0.062 | 0.026 | 0.017 | 1.064 | 1.011 | 1.120 | 0.001 | 0.003 | 0.585 | 0.191 | 51.978 | 0.164 |
|  | MR Egger | | 44 | 0.043 | 0.031 | 0.177 | 1.044 | 0.982 | 1.110 |  |  |  |  |  |  |
|  | Weighted mode | | 44 | 0.063 | 0.025 | 0.015 | 1.065 | 1.015 | 1.119 |  |  |  |  |  |  |
|  | IVW | | 44 | 0.055 | 0.022 | 0.014* | 1.056 | 1.011 | 1.104 |  |  |  |  |  |  |

**Abbreviations:** BMD: bone mineral density; eBMD: estimated BMD; FA: forearm; FN: femoral neck; LS: lumbar; SE: standard error of beta; IVW: Inverse variance weighted; * *P*<0.05.

**Supplementary Table 9.** MR estimate results of n-6 PUFAs on outcomes.

| **Outcome** | | **Methods** | **Nsnp** | **Beta** | **SE** | ***P* value** | **OR** | **or_lci95** | **or_uci95** | **Horizontal pleiotropy** | | | | **Heterogeneity** | |
| --- | --- | --- | --- | --- | --- | --- | --- | --- | --- | --- | --- | --- | --- | --- | --- |
|  |  |  |  |  |  |  |  |  |  | **MR-Egger regression** | | | **MR-PRESSO** | **Cochran’s *Q*** | ***P* value** |
|  |  |  |  |  |  |  |  |  |  | **Egger intercept** | **SE** | ***P* value** | **Global test *P* value** |  |  |
| eBMD | Weighted median | | 32 | 0.051 | 0.012 | <0.001 | 1.053 | 1.028 | 1.078 | 0.001 | 0.001 | 0.405 | 0.096 | 41.731 | 0.094 |
|  | MR Egger | | 32 | 0.037 | 0.020 | 0.066 | 1.038 | 0.999 | 1.078 |  |  |  |  |  |  |
|  | Weighted mode | | 32 | 0.051 | 0.015 | 0.002 | 1.053 | 1.022 | 1.085 |  |  |  |  |  |  |
|  | IVW | | 32 | 0.052 | 0.009 | <0.001* | 1.053 | 1.034 | 1.072 |  |  |  |  |  |  |
| FA | Weighted median | | 50 | -0.045 | 0.079 | 0.570 | 0.956 | 0.819 | 1.116 | -0.001 | 0.006 | 0.866 | 0.494 | 48.546 | 0.491 |
|  | MR Egger | | 50 | 0.036 | 0.101 | 0.721 | 1.037 | 0.851 | 1.263 |  |  |  |  |  |  |
|  | Weighted mode | | 50 | -0.082 | 0.102 | 0.424 | 0.921 | 0.753 | 1.125 |  |  |  |  |  |  |
|  | IVW | | 50 | 0.021 | 0.051 | 0.673 | 1.022 | 0.925 | 1.128 |  |  |  |  |  |  |
| FN | Weighted median | | 49 | 0.021 | 0.038 | 0.581 | 1.021 | 0.948 | 1.099 | -0.004 | 0.003 | 0.148 | 0.144 | 58.483 | 0.143 |
|  | MR Egger | | 49 | 0.084 | 0.054 | 0.128 | 1.087 | 0.978 | 1.208 |  |  |  |  |  |  |
|  | Weighted mode | | 49 | -0.001 | 0.051 | 0.983 | 0.999 | 0.904 | 1.104 |  |  |  |  |  |  |
|  | IVW | | 49 | 0.015 | 0.027 | 0.584 | 1.015 | 0.962 | 1.071 |  |  |  |  |  |  |
| Frac | Weighted median | | 56 | 0.020 | 0.029 | 0.502 | 1.020 | 0.963 | 1.081 | 0.004 | 0.002 | 0.105 | 0.131 | 66.552 | 0.137 |
|  | MR Egger | | 56 | -0.048 | 0.044 | 0.272 | 0.953 | 0.875 | 1.038 |  |  |  |  |  |  |
|  | Weighted mode | | 56 | -0.022 | 0.040 | 0.586 | 0.979 | 0.905 | 1.058 |  |  |  |  |  |  |
|  | IVW | | 56 | 0.014 | 0.022 | 0.545 | 1.014 | 0.970 | 1.059 |  |  |  |  |  |  |
| LS | Weighted median | | 49 | 0.009 | 0.045 | 0.841 | 1.009 | 0.923 | 1.103 | 0.001 | 0.004 | 0.739 | 0.134 | 58.904 | 0.135 |
|  | MR Egger | | 49 | -0.006 | 0.064 | 0.929 | 0.994 | 0.877 | 1.127 |  |  |  |  |  |  |
|  | Weighted mode | | 49 | -0.004 | 0.059 | 0.941 | 0.996 | 0.886 | 1.118 |  |  |  |  |  |  |
|  | IVW | | 49 | 0.013 | 0.032 | 0.688 | 1.013 | 0.951 | 1.078 |  |  |  |  |  |  |

**Abbreviations:** BMD: bone mineral density; eBMD: estimated BMD; FA: forearm; FN: femoral neck; LS: lumbar; SE: standard error of beta; IVW: Inverse variance weighted; * *P*<0.05.

**Supplementary Table 10.** MR estimate results of the ratio of n-3 fatty acids to total fatty acids on outcomes.

| **Outcome** | | **Methods** | **Nsnp** | **Beta** | **SE** | ***P* value** | **OR** | **or_lci95** | **or_uci95** | **Horizontal pleiotropy** | | | | **Heterogeneity** | |
| --- | --- | --- | --- | --- | --- | --- | --- | --- | --- | --- | --- | --- | --- | --- | --- |
|  |  |  |  |  |  |  |  |  |  | **MR-Egger regression** | | | **MR-PRESSO** | **Cochran’s *Q*** | ***P* value** |
|  |  |  |  |  |  |  |  |  |  | **Egger intercept** | **SE** | ***P* value** | **Global test *P* value** |  |  |
| eBMD | Weighted median | | 21 | 0.031 | 0.017 | 0.070 | 1.031 | 0.997 | 1.066 | 0.001 | 0.001 | 0.552 | 0.085 | 29.346 | 0.081 |
|  | MR Egger | | 21 | 0.015 | 0.024 | 0.544 | 1.015 | 0.968 | 1.065 |  |  |  |  |  |  |
|  | Weighted mode | | 21 | 0.018 | 0.021 | 0.399 | 1.018 | 0.977 | 1.061 |  |  |  |  |  |  |
|  | IVW | | 21 | 0.028 | 0.013 | 0.035* | 1.028 | 1.002 | 1.055 |  |  |  |  |  |  |
| FA | Weighted median | | 37 | 0.102 | 0.041 | 0.012 | 1.108 | 1.022 | 1.200 | -0.003 | 0.005 | 0.507 | 0.373 | 39.628 | 0.311 |
|  | MR Egger | | 37 | 0.106 | 0.049 | 0.037 | 1.112 | 1.010 | 1.223 |  |  |  |  |  |  |
|  | Weighted mode | | 37 | 0.093 | 0.042 | 0.034 | 1.098 | 1.010 | 1.193 |  |  |  |  |  |  |
|  | IVW | | 37 | 0.086 | 0.038 | 0.025* | 1.090 | 1.011 | 1.174 |  |  |  |  |  |  |
| FN | Weighted median | | 36 | 0.001 | 0.019 | 0.962 | 1.001 | 0.963 | 1.040 | <0.001 | 0.003 | 0.894 | 0.091 | 55.039 | 0.017 |
|  | MR Egger | | 36 | -0.010 | 0.028 | 0.729 | 0.990 | 0.937 | 1.047 |  |  |  |  |  |  |
|  | Weighted mode | | 36 | -0.003 | 0.020 | 0.890 | 0.997 | 0.959 | 1.037 |  |  |  |  |  |  |
|  | IVW | | 36 | -0.008 | 0.022 | 0.732 | 0.992 | 0.951 | 1.036 |  |  |  |  |  |  |
| Frac | Weighted median | | 37 | -0.064 | 0.017 | <0.001 | 0.938 | 0.907 | 0.970 | 0.002 | 0.002 | 0.512 | 0.097 | 55.675 | 0.019 |
|  | MR Egger | | 37 | -0.058 | 0.024 | 0.020 | 0.943 | 0.900 | 0.988 |  |  |  |  |  |  |
|  | Weighted mode | | 37 | -0.062 | 0.017 | 0.001 | 0.940 | 0.909 | 0.973 |  |  |  |  |  |  |
|  | IVW | | 37 | -0.049 | 0.019 | 0.009* | 0.953 | 0.918 | 0.988 |  |  |  |  |  |  |
| LS | Weighted median | | 36 | 0.051 | 0.023 | 0.027 | 1.053 | 1.006 | 1.102 | -0.004 | 0.004 | 0.234 | 0.052 | 68.846 | <0.001 |
|  | MR Egger | | 36 | 0.052 | 0.036 | 0.160 | 1.053 | 0.981 | 1.130 |  |  |  |  |  |  |
|  | Weighted mode | | 36 | 0.052 | 0.025 | 0.040 | 1.054 | 1.004 | 1.106 |  |  |  |  |  |  |
|  | IVW | | 36 | 0.025 | 0.029 | 0.384 | 1.025 | 0.969 | 1.085 |  |  |  |  |  |  |

**Abbreviations:** BMD: bone mineral density; eBMD: estimated BMD; FA: forearm; FN: femoral neck; LS: lumbar; SE: standard error of beta; IVW: Inverse variance weighted; * *P*<0.05.

**Supplementary Table 11.** MR estimate results of the ratio of n-6 PUFAs to n-3 PUFAs on outcomes.

| **Outcome** | | **Methods** | **Nsnp** | **Beta** | **SE** | ***P* value** | **OR** | **or_lci95** | **or_uci95** | **Horizontal pleiotropy** | | | | **Heterogeneity** | |
| --- | --- | --- | --- | --- | --- | --- | --- | --- | --- | --- | --- | --- | --- | --- | --- |
|  |  |  |  |  |  |  |  |  |  | **MR-Egger regression** | | | **MR-PRESSO** | **Cochran’s *Q*** | ***P* value** |
|  |  |  |  |  |  |  |  |  |  | **Egger intercept** | **SE** | ***P* value** | **Global test *P* value** |  |  |
| eBMD | Weighted median | | 20 | -0.057 | 0.015 | <0.001 | 0.944 | 0.916 | 0.973 | <0.001 | 0.001 | 0.887 | 0.175 | 25.791 | 0.136 |
|  | MR Egger | | 20 | -0.057 | 0.025 | 0.034 | 0.944 | 0.899 | 0.992 |  |  |  |  |  |  |
|  | Weighted mode | | 20 | -0.065 | 0.019 | 0.003 | 0.937 | 0.903 | 0.973 |  |  |  |  |  |  |
|  | IVW | | 20 | -0.054 | 0.012 | <0.001* | 0.947 | 0.924 | 0.970 |  |  |  |  |  |  |
| FA | Weighted median | | 32 | -0.109 | 0.044 | 0.014 | 0.897 | 0.822 | 0.978 | 0.003 | 0.005 | 0.569 | 0.185 | 40.335 | 0.122 |
|  | MR Egger | | 32 | -0.116 | 0.056 | 0.048 | 0.891 | 0.798 | 0.994 |  |  |  |  |  |  |
|  | Weighted mode | | 32 | -0.096 | 0.040 | 0.022 | 0.908 | 0.839 | 0.982 |  |  |  |  |  |  |
|  | IVW | | 32 | -0.095 | 0.042 | 0.025* | 0.910 | 0.837 | 0.988 |  |  |  |  |  |  |
| FN | Weighted median | | 33 | -0.001 | 0.021 | 0.970 | 0.999 | 0.958 | 1.042 | -0.001 | 0.003 | 0.735 | 0.054 | 50.008 | 0.022 |
|  | MR Egger | | 33 | 0.008 | 0.029 | 0.786 | 1.008 | 0.952 | 1.068 |  |  |  |  |  |  |
|  | Weighted mode | | 33 | 0.007 | 0.020 | 0.745 | 1.007 | 0.967 | 1.048 |  |  |  |  |  |  |
|  | IVW | | 33 | 0.002 | 0.022 | 0.945 | 1.002 | 0.959 | 1.046 |  |  |  |  |  |  |
| Frac | Weighted median | | 32 | 0.036 | 0.042 | 0.394 | 1.037 | 0.954 | 1.127 | 0.001 | 0.003 | 0.802 | 0.472 | 31.366 | 0.448 |
|  | MR Egger | | 32 | 0.022 | 0.060 | 0.724 | 1.022 | 0.908 | 1.150 |  |  |  |  |  |  |
|  | Weighted mode | | 32 | 0.003 | 0.054 | 0.953 | 1.003 | 0.903 | 1.114 |  |  |  |  |  |  |
|  | IVW | | 32 | 0.035 | 0.029 | 0.225 | 1.035 | 0.979 | 1.095 |  |  |  |  |  |  |
| LS | Weighted median | | 32 | 0.057 | 0.024 | 0.020 | 1.058 | 1.009 | 1.110 | 0.003 | 0.003 | 0.280 | 0.151 | 43.414 | 0.068 |
|  | MR Egger | | 32 | 0.046 | 0.032 | 0.165 | 1.047 | 0.983 | 1.115 |  |  |  |  |  |  |
|  | Weighted mode | | 32 | 0.058 | 0.026 | 0.032 | 1.060 | 1.008 | 1.115 |  |  |  |  |  |  |
|  | IVW | | 32 | 0.069 | 0.025 | 0.005* | 1.071 | 1.021 | 1.124 |  |  |  |  |  |  |

**Abbreviations:** BMD: bone mineral density; eBMD: estimated BMD; FA: forearm; FN: femoral neck; LS: lumbar; SE: standard error of beta; IVW: Inverse variance weighted; * *P*<0.05.

**Supplementary Table 12.** MR estimate results of the circulating level of cytokines on PUFAs.

| **Exposure** | **Outcome** | **Methods** | **Nsnp** | **Beta** | **SE** | ***P* value** | **OR** | **or_lci95** | **or_uci95** | **Horizontal pleiotropy** | | |  |  | |
| --- | --- | --- | --- | --- | --- | --- | --- | --- | --- | --- | --- | --- | --- | --- | --- |
|  |  |  |  |  |  |  |  |  |  | **MR-Egger regression** | | | **MR-PRESSO** | **Cochran’s *Q*** | ***P* value** |
|  |  |  |  |  |  |  |  |  |  | **Egger intercept** | **SE** | ***P* value** | **Global test *P* value** |  |  |
| IL-6 | n-3 PUFAs | IVW | 2 | -0.034 | 0.047 | 0.474 | 0.967 | 0.882 | 1.060 |  |  |  |  | 2.171 | 0.141 |
| IL-6 | n-3 pct | IVW | 2 | -0.059 | 0.032 | 0.065 | 0.943 | 0.886 | 1.004 |  |  |  |  | 0.553 | 0.457 |
| IL-6 | n-6 PUFAs | IVW | 2 | 0.016 | 0.057 | 0.773 | 1.016 | 0.910 | 1.136 |  |  |  |  | 3.220 | 0.073 |
| IL-6 | n-6 to n-3 | IVW | 2 | 0.046 | 0.032 | 0.147 | 1.047 | 0.984 | 1.115 |  |  |  |  | 0.923 | 0.337 |
|  |  |  |  |  |  |  |  |  |  |  |  |  |  |  |  |
| TNFb | n-3 PUFAs | IVW | 2 | 0.006 | 0.008 | 0.458 | 1.006 | 0.990 | 1.022 |  |  |  |  | 0.512 | 0.474 |
| TNFb | n-3 pct | IVW | 2 | -0.001 | 0.009 | 0.910 | 0.999 | 0.981 | 1.017 |  |  |  |  | 1.380 | 0.240 |
| TNFb | n-6 PUFAs | IVW | 2 | 0.002 | 0.008 | 0.825 | 1.002 | 0.986 | 1.017 |  |  |  |  | 0.679 | 0.410 |
| TNFb | n-6 to n-3 | IVW | 2 | -0.006 | 0.008 | 0.477 | 0.994 | 0.978 | 1.010 |  |  |  |  | 1.085 | 0.298 |
|  |  |  |  |  |  |  |  |  |  |  |  |  |  |  |  |
| BMP-7 | n-3 PUFAs | IVW | 2 | -0.029 | 0.016 | 0.073 | 0.971 | 0.941 | 1.003 |  |  |  |  | 0.024 | 0.877 |
| BMP-7 | n-3 pct | IVW | 2 | -0.034 | 0.016 | 0.038* | 0.967 | 0.937 | 0.998 |  |  |  |  | 0.010 | 0.921 |
| BMP-7 | n-6 PUFAs | IVW | 2 | 0.002 | 0.016 | 0.914 | 1.002 | 0.971 | 1.034 |  |  |  |  | 0.471 | 0.493 |
| BMP-7 | n-6 to n-3 | IVW | 2 | 0.034 | 0.016 | 0.036* | 1.035 | 1.002 | 1.069 |  |  |  |  | 0.161 | 0.689 |

**Abbreviations:** PUFAs: polyunsaturated fatty acids; n-3 pct: the ratio of n-3 fatty acids to total fatty acids; n-6 to n-3: the ratio of n-6 PUFAs to n-3 PUFAs; IL: interleukin; TNF: Tumor necrosis factor; BMP: bone morphogenetic protein; SE: standard error of beta; IVW: Inverse variance weighted; * *P*<0.05.

**Supplementary Table 13.** MR estimate results of the circulating level of cytokines on BMD.

| **Exposure** | **Outcome** | **Methods** | **Nsnp** | **Beta** | **SE** | ***P* value** | **OR** | **or_lci95** | **or_uci95** | **Horizontal pleiotropy** | | | | **Heterogeneity** | |
| --- | --- | --- | --- | --- | --- | --- | --- | --- | --- | --- | --- | --- | --- | --- | --- |
|  |  |  |  |  |  |  |  |  |  | **MR-Egger regression** | | | **MR-PRESSO** | **Cochran’s *Q*** | ***P* value** |
|  |  |  |  |  |  |  |  |  |  | **Egger intercept** | **SE** | ***P* value** | **Global test *P* value** |  |  |
| IL-6 | eBMD | Wald ratio | 1 | 0.014 | 0.019 | 0.448 | 1.014 | 0.978 | 1.052 |  |  |  |  |  |  |
| IL-6 | FA | IVW | 2 | -0.094 | 0.121 | 0.436 | 0.910 | 0.719 | 1.153 |  |  |  |  | 0.047 | 0.829 |
| IL-6 | FN | IVW | 2 | 0.060 | 0.059 | 0.317 | 1.061 | 0.945 | 1.192 |  |  |  |  | 0.610 | 0.435 |
| IL-6 | Frac | IVW | 2 | -0.014 | 0.075 | 0.851 | 0.986 | 0.851 | 1.142 |  |  |  |  | 2.171 | 0.141 |
| IL-6 | LS | IVW | 2 | 0.037 | 0.069 | 0.590 | 1.038 | 0.906 | 1.189 |  |  |  |  | 0.460 | 0.498 |
|  |  |  |  |  |  |  |  |  |  |  |  |  |  |  |  |
| TNF-b | eBMD | IVW | 2 | 0.005 | 0.005 | 0.374 | 1.005 | 0.995 | 1.015 |  |  |  |  | 2.092 | 0.148 |
| TNF-b | FA | IVW | 2 | 0.010 | 0.031 | 0.746 | 1.010 | 0.950 | 1.074 |  |  |  |  | 1.117 | 0.291 |
| TNF-b | FN | IVW | 2 | -0.009 | 0.015 | 0.565 | 0.991 | 0.963 | 1.021 |  |  |  |  | 1.038 | 0.308 |
| TNF-b | Frac | IVW | 2 | 0.002 | 0.021 | 0.938 | 1.002 | 0.960 | 1.045 |  |  |  |  | 2.868 | 0.090 |
| TNF-b | LS | IVW | 2 | 0.011 | 0.019 | 0.583 | 1.011 | 0.973 | 1.050 |  |  |  |  | 1.282 | 0.258 |
|  |  |  |  |  |  |  |  |  |  |  |  |  |  |  |  |
| BMP-7 | eBMD | IVW | 2 | -0.009 | 0.015 | 0.545 | 0.991 | 0.961 | 1.021 |  |  |  |  | 4.326 | 0.038 |
| BMP-7 | FA | IVW | 2 | -0.024 | 0.062 | 0.703 | 0.977 | 0.865 | 1.103 |  |  |  |  | 0.260 | 0.610 |
| BMP-7 | FN | IVW | 2 | 0.014 | 0.031 | 0.639 | 1.014 | 0.955 | 1.077 |  |  |  |  | 0.003 | 0.957 |
| BMP-7 | Frac | IVW | 2 | 0.013 | 0.026 | 0.606 | 1.014 | 0.963 | 1.067 |  |  |  |  | 0.490 | 0.484 |
| BMP-7 | LS | IVW | 2 | -0.005 | 0.035 | 0.881 | 0.995 | 0.928 | 1.066 |  |  |  |  | 0.016 | 0.901 |

**Abbreviations:** BMD: bone mineral density; eBMD: estimated BMD; FA: forearm; FN: femoral neck; LS: lumbar; interleukin; IL: interleukin; TNF: Tumor necrosis factor; BMP: bone morphogenetic protein; SE: standard error of beta; IVW: Inverse variance weighted.
